# Robust inference with GhostKnockoffs in genome-wide association studies

**DOI:** 10.21203/rs.3.rs-6396196/v1

**Published:** 2025-05-05

**Authors:** Xinran Qi, Michael E. Belloy, Jiaqi Gu, Xiaoxia Liu, Hua Tang, Zihuai He

**Affiliations:** 1 Department of Hematology and Hematopoietic Cell Transplantation, City of Hope Comprehensive Cancer Center, Duarte, CA 91010, USA; 2 Department of Neurology, Washington University in Saint Louis, Saint Louis, MO 63108, USA; 3 Department of Neurology and Neurological Sciences, Stanford University, Stanford, CA 94305, USA; 4 Department of Genetics, Stanford University, Stanford, CA 94305, USA; 5 Quantitative Sciences Unit, Department of Medicine, Stanford University, Stanford, CA 94305, USA; 6 Department of Biomedical Data Science, Stanford University, Stanford, CA 94305, USA; 7 Lead contact

## Abstract

Genome-wide association studies (GWASs) have been extensively adopted to depict the underlying genetic architecture of complex traits. Recent studies have demonstrated that for feature selection in GWASs data, in addition to controlling the familywise error rate (FWER), the false discovery rate (FDR) serves as an appealing alternative for detecting small effect loci associated with polygenic traits. However, the presence of correlations among genetic variants makes direct application of usual FDR-controlling procedures to marginal association tests ineffective. The knockoffs-based methods have shown guarantee in FDR control in GWASs, but their statistical validity and effectiveness in studies with related individuals remain unexplored. In this paper, we propose a knockoff-based approach by integrating recently proposed GhostKnockoffs and state-of-the-art marginal association tests. We show that GhostKnockoffs, which only requires GWAS Z-scores as input, is robust to arbitrary relatedness structure as long as the input Z-scores are derived from valid generalized linear mixed models. Therefore, it can be flexibly applied on top of the standard GWASs pipeline that accounts for relatedness to enhance the discovery of small effect loci. This robustness also generalizes GhostKnockoffs to other GWASs settings, such as the meta-analysis of multiple overlapping studies and studies based on association test statistics deviated from score tests. We demonstrate the method’s performance using simulation studies and a meta-analysis of nine European ancestral genome-wide association studies and whole exome/genome sequencing studies for the Alzheimer’s disease.

## Introduction

Genome-wide association studies (GWASs) have been extensively adopted to depict the underlying genetic architecture of complex traits. According to the last data release on 2025-3-8, the National Human Genome Research Institute-European Bioinformatics Institute (NHGRI-EBI) Catalog of human GWASs^1^ contains 7,190 publications, 788,652 top genotype-phenotype associations, and 112,377 full summary statistics. Despite the considerable success, GWASs based on marginal association tests encounter challenges in the post-GWASs era. While a large proportion of heritability remains unexplained, there is growing evidence that supports the high polygenicity of many complex traits. A multitude of small effect risk loci that currently lie below the stringent threshold which controls for family-wise error rate (FWER) can be informative to understand complex traits and to enhance risk predictions. The more recent omnigenic model^2^ also supports lines of research from discovering “isolated” variant-gene-phenotype triplets to probing into pathways and regulations among variants with infinitesimal effect that influence complex phenotypes. Current studies have demonstrated that for feature selection in GWASs data, in addition to FWER control, the false discovery rate (FDR) serves as an effective alternative for detecting small effect loci associated with polygenic traits. However, the Benjamini-Hochberg (BH) procedure for FDR control may encounter challenges in accounting for the correlations among marginal tests in GWASs. In addition, the presence of closely located SNPs reflecting the same biological association complicates the definition and counting of discoveries. This makes it unclear how FDR should be properly defined in such settings^3^.

In recent years, the model-*X* knockoffs inference^4–9^ has been a powerful statistical framework to identify putative causal genetic variants for complex phenotypes. Complementary to GWASs testing for marginal genotype-phenotype associations and controlling for FWER, the knockoffs inference performs high-dimensional conditional independent tests and controls for FDR, with corresponding null hypothesis “phenotype *Y_i_* is independent of genotype *G_ij_* given remaining genotypes ***G***_*i*,−***j***_”, where *i* ∈ {1, …, *n*} denotes sample index and *j* ∈ {1, …, *p*} denotes variant index. The conditional independent test is done by generating knockoffs counterparts exchangeable to original genetic variants but are conditionally independent of phenotypes given original variants. Knockoffs counterparts act as negative controls in contrast to original variants to account for the LD pattern while measuring variants’ importance for the phenotypes. Previous studies show that variants identified by knockoffs inference are more likely to be causal. The FDR control also allows for additional discoveries of variants with weaker effect that are missed by conventional GWASs, especially for complex traits with a polygenic/omnigenic genetic architecture^4–9^.

While the existence of sample relatedness is common in recent large-scale genetic association studies^10–14^, most knockoffs-based methods are proposed for studies with independent samples. Bates et al. (2020)^15^ and Yang et al. (2022)^16^ proposed methods for studies with known pedigree structure (e.g. studies with trio design). Sesia et al. (2021)^7^ proposed a method that controls for unknown diverse ancestries or familial relatedness by constructing knockoffs counterparts that retain sample relatedness. However, the proposed knockoffs construction is a highly nontrivial task and can be computationally intensive^6; 9^, which requires individual-level data to estimate haplotypes, estimate relatedness graph, and partition related samples into independent identity-by-descent (IBD)-sharing families.

In this paper, we propose a simple and effective knockoffs-based method to account for sample relatedness, which primarily requires GWASs summary statistics. As noted by Sesia et al. (2021)^7^, the robustness of knockoffs inference in the presence of sample relatedness presents a significant challenge. Our proposed method is based on the recently proposed GhostKnockoffs^9^, which perform knockoffs inference without the need to generate individual-level knockoffs for hundreds of thousands of samples. We show in both theory and in simulation studies that GhostKnockoffs with input marginal Z-scores based on commonly used generalized linear mixed models can efficiently control FDR for related samples. In general, we show that GhostKnockoffs inference is robust to its input Z-scores as long as they are from valid marginal association tests and their correlations are consistent with the correlations among the corresponding genetic variants. This appealing property naturally extends GhostKnockoffs to many other useful settings in GWASs, such as the meta-analysis of multiple overlapping studies and studies based on test statistics deviated from conventional score tests. On the other hand, the vanilla knockoffs based on individual-level data with the same appropriate “upstream” GWASs summary statistics do not necessarily control FDR.

We applied GhostKnockoffs to the meta-analysis of nine European ancestral genome-wide association studies and whole exome/genome sequencing studies to select putative causal genetic variants for the Alzheimer’s disease (AD). We showed that the proposed method identified 41 loci at FDR=0.1, including 23 loci that are missed by conventional marginal association tests with FWER control. We leveraged the Open Targets Genetics Variant-to-Gene (V2G) strategy to link the selected variants to functionally relevant genes. We additionally applied other variant-to-gene mapping strategies such as proximal gene mapping, and the optimal combined SNP-to-gene (cS2G) strategy^17^. Finally, we utilized single-cell RNA sequences (scRNAseq) consisting of 143,793 single-nucleus transcriptomes from two human brain components, hippocampus (9 AD cases and 8 controls) and cortex (4 AD cases and 4 controls) to validate genes mapped by the Open Targets V2G and other strategies.

## Methods

### Overview

**Figure 1:**
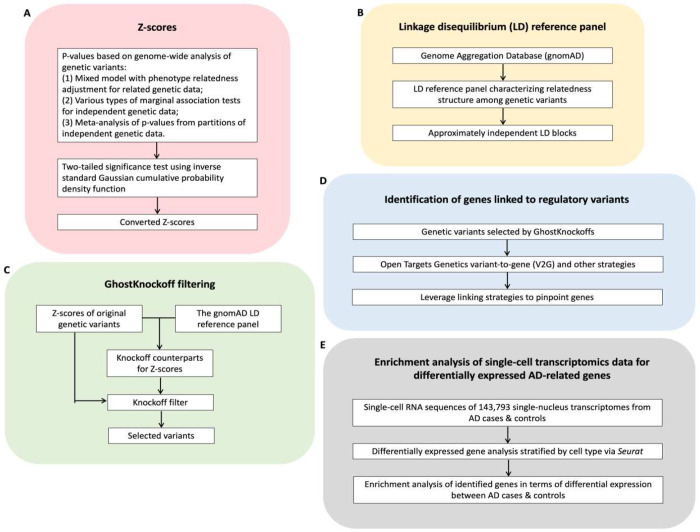
Overview of the analysis workflow. Schematic of the proposed GhostKnockoffs approach based on summary statistics. **A.** Input Z-scores can be derived from GWAS summary statistics. **B.** Linkage disequilibrium (LD) matrix can be obtained from a reference panel. **C.** GhostKnockoffs inference using Z-scores and LD reference panel as input. **D.** Prioritize target genes of the identified variants. **E.** Downstream analysis of transcriptomics data to validate the enrichment of biological function of the mapped genes.

We propose a knockoffs inference method to perform genome-wide conditional independent tests in human genetic studies based on summary statistics (e.g., p-values/Z-scores, directions of effect, and reference panel LD). The proposed method integrates recent advances in knockoffs statistics, namely GhostKnockoffs, to improve power and to prioritize putative causal variants, generalized linear mixed effect model to control for sample relatedness, and meta-analysis strategies to aggregate multiple studies allowing for arbitrary sample overlap. The workflow is presented in Figure 1.

As shown in Figure 1A, our proposed method takes pre-calculated p-values and directions of effect as input. The p-values can be calculated from various types of marginal association tests whose embedded assumptions suit the data structure, such as the generalized linear mixed effect model to account for sample relatedness, saddle point approximation for extreme case-control imbalance and meta-analysis that aggregates multiple studies. The p-values and directions of effect are then converted to corresponding Z-scores of a two-tailed significance test using the inverse normal transformation.

In contrast to conventional GWASs which perform marginal association tests to evaluate whether a variant is associated with the phenotypes, our method utilizes a LD reference panel such as the Genome Aggregation Database (gnomAD)^18^ to perform conditional tests evaluating whether a variant is associated with the phenotypes conditioning on other variants in the same LD block (Figure 1B). The conditional tests naturally reduce the effect of LD, and thereby each of the associated loci includes fewer genetic variants on average^9^.

The pre-calculated Z-scores and LD reference panel serve as the input of the recently proposed GhostKnockoffs. As shown in Figure 1C, GhostKnockoffs generate multiple knockoffs counterparts of the original Z-scores. The GhostKnockoffs’ feature importance scores can be measured by the square of original Z-scores and knockoffs counterparts’ Z-scores. The gap of feature importance scores between original genetic variants and corresponding knockoffs counterparts quantifies the association between variants and phenotypes and is later compared with a data-specific threshold controlling FDR to select putative casual variants.

We then leverage and compare external functional information to further pinpoint mechanism-based target genes of the identified variants and to discover the underlying genetic structure of biological functions using the Open Targets Genetics variant-to-gene (V2G) strategy (Figure 1D). The Open Targets Genetics V2G pipeline systematically integrates evidence from molecular QTLs (eQTLs, pQTLs, sQTLs), chromatin interaction experiments (e.g., Promoter Capture Hi-C), in silico functional predictions (e.g., Variant Effect Predictor), and proximity to gene transcription start sites to prioritize variant-gene associations.

Finally, we perform downstream analysis of transcriptomics data to validate enrichment of biological functions of the mapped genes (Figure 1E) using single-cell RNA-sequencing data. In this particular data analysis for the AD, we use 143,793 single-nucleus transcriptomes from 17 hippocampus (8 controls and 9 AD cases) and 8 cortex samples (4 controls and 4 AD cases)^19^. We compare the enrichment analysis of single-cell transcriptomics data for AD-related genes identified by the V2G and other strategies (cS2G and proximal genes) based on the proposed method’s variable selection results and those of conventional GWASs.

### P-value calculation via generalized linear mixed model (GLMM) and score test

While the proposed method can take p-values from various types of marginal association tests as input, we use the commonly used generalized linear mixed model as an example to describe the method. Consider a study with *n* possibly related participants, *p* genetic variants and *q* other covariates. The relationship between participants is not assumed to be known. For the *i^th^* individual, the scalar *Y_i_* denotes its phenotype measurement (either quantitative or dichotomous); the 1 × *p* row vector ***G_i_*** = (*G*_*i*1_, …, *G_ip_*) ∈ {0,1,2}*^p^* denotes genotypes of genetic variants; the 1 × *q* row vector ***X_i_*** = (*X*_*i*1_, …, *X_iq_*) denotes other covariates. The marginal GLMM for quantitative/dichotomous phenotype and variant *k* is:

$$g\left(\mu_{ik}\right)=X_{i}\alpha_{k}+G_{ik}\beta_{k}+b_{ik},$$

where $\mu_{ik}=E\left(y_{i}|X_{i},G_{ik},b_{ik}\right)$ denotes the expectation of the *i^th^* phenotype *y_i_*, conditional on covariate ***X_i_***, genotype *G_ik_* and random effect *b_ik_*. The *p* × 1 column vector $\beta_{i}={\left(\beta_{i1},\cdots ,\beta_{ip}\right)}^{T}$ denotes genotype effects. The *q* × 1 column vector $\alpha_{k}={\left(\alpha_{k1},\cdots ,\alpha_{kq}\right)}^{T}$ denotes fixed covariate effect plus intercept. The link function is set to be *g*(*x*) = *x* for a quantitative normally distributed phenotype and *g*(*x*) = logit(*x*) for a dichotomous phenotype. The *n* × 1 column vector $b_{k}={\left(b_{ik}\right)}^{T}∼N\left(0,\theta_{k}\Phi \right)$ denotes random effect with the variance component parameter $\theta_{k}$ and the *n* × *n* matrix Φ measuring sample relatedness.

The Z-scores based on single-variant score test p-values, denoted as $Z_{\text{score},k}=sign\left(\beta_{k}\right)\Phi^{-1}\left(\frac{p-{\text{value}}_{k}}{2}\right)$, (see “Supplemental Materials” for derivations) are utilized for the next steps.

### The gnomAD LD reference panel to facilitate conditional test

Our method utilizes the European LD reference panel from the Genome Aggregation Database (gnomAD)^18^ to perform conditional tests (Figure 1B) in the current analysis because our simulated data represents the European population. The gnomAD estimates LD more accurately by aggregating worldwide large-scale sequencing projects’ exome and genome sequencing data. It surpasses the 1000 Genomes Project whose majority samples of sequencing data are included in gnomAD and hence makes more robust estimates of the LD reference panel. Based on the gnomAD LD estimates, we further partition variants into 1703 approximately independent linkage disequilibrium blocks defined by Berisa and Pickrell (2016)^20^.

### GhostKnockoffs inference based on Z-scores

As opposed to typical model-X knockoffs which require individual-level genotype data, GhostKnockoffs directly use the Z-scores as input to generate multiple knockoffs counterparts by

(1) $${\tilde{Z}}_{\text{score}}=PZ_{\text{score}}+E,E∼N(0,V)$$

where the *pM* × 1 column vector ${\tilde{Z}}_{\text{score}}={\left({\tilde{Z}}_{\text{score}}^{m}\right)}_{1\leq m\leq M}$ consists of Z-scores for *M* knockoffs counterparts per genetic variant. The *pM* × *p* matrix ***P*** and *pM* × *pM* matrix ***V*** are defined by the input LD matrix such that the inference based on summary statistics is equivalent to the multiple second-order knockoffs inference based on individual-level data for independent samples^9^. Intuitively, matrix ***P*** serves as a projection matrix based on LD such that ${\tilde{Z}}_{\text{score}}$ quantifies the indirect effect through correlation with other variants. The corresponding feature importance scores $T^{m}={\left({\tilde{Z}}_{\text{score}}^{m}\right)}^{2}$ are compared with $T={\left(Z_{\text{scores}}\right)}^{2}$, refered to as

$$W=(T-\underset{1\leq m\leq M}{\text{median}}T^{m})I_{T\geq \underset{1\leq m\leq M}{\max}T^{m}},$$

to quantify the association between variants and the phenotypes, conditioning on other variants in the same LD block. Based on the user-specified FDR level, a threshold τ is chosen and genetic variants whose W> τ are selected by GhostKnockffs.

While the GhostKnockoffs method is originally proposed for independent samples, we demonstrate its robustness to input summary statistics via extensive simulation studies. Specifically, we show that the GhostKnockoffs inference remains valid if the input p-values are correctly calculated. We also discussed the conditions under which theoretical robustness can be achieved in “Supplemental Materials”.

### Simulate unrelated & related genotypes

We simulate unrelated and related genotypes based on the haplotype dataset from the R package *SKAT*^21^, which consists of 10,000 haplotypes over 200K BP region and is generated from the calibration coalescent model (COSI) software and mimics the LD structure of the European ancestry^20; 22^. Unrelated genotypes are simulated based on randomly sampled haplotypes. Related genotypes are simulated using the three-generation pedigree, as shown in Figure S11. For each family that consists of 10 family members, two unrelated grandparents are simulated based on randomly sampled haplotypes. Their offspring genotypes (the second-generation dependent parents) are simulated using the gene dropping algorithm. Given that the *SKAT* haplotypes dataset consists of haplotypes over 200K base pair region, the recombination rate is set to be 0 while simulating offspring genotypes. Two other unrelated second-generation parents are randomly sampled from the *SKAT* haplotypes dataset similarly to pair with the second-generation dependent parents correspondingly. The third-generation offspring genotypes are simulated in a similar way using the gene dropping algorithm.

We simulate genotypes of (1) 10,000 unrelated individuals and (2) 1000 families according to the pedigree in Figure S11. 1000 genetic variants are randomly selected, out of which variants whose MAF greater than 0.01 are kept for the robust calculation of marginal association test p-values. We apply the single linkage hierarchical clustering to variants and group them to clusters so that there are no two clusters having cross-correlation above 0.75. A representative variant with the largest sum of absolute values of within-correlations is selected per cluster for simulation study.

### Simulate unrelated & related phenotypes

We randomly select 10 causal variants to simulate quantitative and dichotomous phenotypes. The absolute value of regression coefficient for the *j^th^* causal variant is $\left|\beta_{j}\right|=\sqrt{\frac{a}{10\times Var\left({G.}_{j}\right)}}$, where *j* ∈ {1,…,1000}. The hyperparameter *a* denotes the variance explained by causal variants and is a constant. For quantitative phenotypes, *a* = 1 and for dichotomous traits, *a* = 2.5. We define that half of causal variants show protective effect (negative sign of $\beta_{j}$) and the other half show risk effect (positive sign $\beta_{j}$).

#### Unrelated phenotypes.

We simulate independent phenotypes based on unrelated genotypes ***G*** as follows.

Linear fixed effect model for quantitative phenotype *Y_i_*:

$$Y_{i}=X_{i1}+G_{i}\beta +\epsilon_{i}^{Q}.$$

Logistic fixed effect model for dichotomous phenotype *Y_i_*:

$$\text{logit}\left(\pi_{i}\right)=\beta_{0}+X_{i1}+G_{i}\beta +\epsilon_{i}^{D},$$

where ***G_i_*** = (*G_ij_*)^T^ denotes genotypes for the *i^th^* individual, *i* ∈ {1,…,10000}. *X*_*i*1_~*N*(0,1) denotes the fixed effect covariate. $\epsilon_{i}^{Q}∼N(0,\sqrt{8})$ denotes the residual of the linear model. $\epsilon_{i}^{D}∼N(0,1)$ measures the variation due to unobserved covariates of the logistic model. *X*_*i*1_, $\epsilon_{i}^{Q}$ and $\epsilon_{i}^{D}$ are mutually independent. $\beta_{0}$ denotes the intercept of the logistic model corresponding to prevalence being 10%. The link function is $logit\left(\pi_{i}\right)=log\left(\frac{\pi_{i}}{1-\pi_{i}}\right)$. The $\pi_{i}=Pr\left(Y_{i}=1|X_{i1},G_{i}\right)$ denotes the conditional mean of *Y_i_* given covariate *X*_*i*1_ and genotypes ***G_i_***.

#### Related phenotypes.

We simulate related phenotypes based on related genotypes as follows.

Linear mixed effect model for quantitative phenotype *Y_i_*:

$$Y_{i}=X_{i1}+G_{i}\beta +b_{i}+\epsilon_{i}^{Q}.$$

Logistic mixed effect model for dichotomous phenotype *Y_i_*:

$$\text{logit}\left(\pi_{i}\right)=\beta_{0}+X_{i1}+G_{i}\beta +b_{i},$$

where *b_i_* denotes the random effect and b∼N(0,Σ). The Σ=θΦ denotes the variance covariance matrix of random effects, where θ is the variance component parameter and Φ denotes the kinship matrix corresponding to the three-generation pedigree.


$$\Phi =\left[\begin{array}{cccccccccc} 1 & 0 & 0.5 & 0.5 & 0 & 0 & 0.25 & 0.25 & 0.25 & 0.25\\ 0 & 1 & 0.5 & 0.5 & 0 & 0 & 0.25 & 0.25 & 0.25 & 0.25\\ 0.5 & 0.5 & 1 & 0.5 & 0 & 0 & 0.5 & 0.5 & 0.25 & 0.25\\ 0.5 & 0.5 & 0.5 & 1 & 0 & 0 & 0.25 & 0.25 & 0.5 & 0.5\\ 0 & 0 & 0 & 0 & 1 & 0 & 0.5 & 0.5 & 0 & 0\\ 0 & 0 & 0 & 0 & 0 & 1 & 0 & 0 & 0.5 & 0.5\\ 0.25 & 0.25 & 0.5 & 0.25 & 0.5 & 0 & 1 & 0.5 & 0.125 & 0.125\\ 0.25 & 0.25 & 0.5 & 0.25 & 0.5 & 0 & 0.5 & 1 & 0.125 & 0.125\\ 0.25 & 0.25 & 0.25 & 0.5 & 0 & 0.5 & 0.125 & 0.125 & 1 & 0.5\\ 0.25 & 0.25 & 0.25 & 0.5 & 0 & 0.5 & 0.125 & 0.125 & 0.5 & 1 \end{array}\right].$$

For linear mixed model, we set the sum of variance due to residual *∊^Q^* and random effects ***b*** as a constant value. Therefore, $\epsilon_{i}^{Q}∼N\left(0,\sqrt{Var\left(\epsilon^{Q}\right)}\right)$, where Var(*∊^Q^*) = 8 – Var(***b***) so that we guarantee the power at a similar level while changing the value of variance component parameter θ∈{1,4,7}. For logistic mixed effect model, the variance component parameter θ also varies within the range θ∈{1,4,7}.

### Case control family study for related dichotomous phenotypes

We design three case-control family studies for dichotomous phenotypes to compare GhostKnockoffs with the second-order knockoffs under different levels of sample relatedness. The parameter $K=\left(\frac{\sum_{i,j}d(i,j)\times f(i,j)}{\#\text{of}(i,j)\text{pairs in family}}\right)$/(#of families) measures the sample relatedness of phenotypes from each sampling scheme. For each pair of individuals, *d*(*i, j*) = 1 if both are cases/controls and *d*(*i, j*) = 0 otherwise; *f*(*i, j*) = 1 if both are from the same family and *f*(*i, j*) = 0 otherwise.

The following three sampling schemes are based on related genotypes simulated using the gene dropping algorithm and related phenotypes simulated using the logistic mixed effect model, which we abbreviate as the simulated foundation data. Each sample scheme has 10,000 individuals.

***Scheme A*** We randomly select 5000 cases (*Y* = 1) and 5000 controls (*Y* = 0) from the simulated foundation data. (*K*=0.383)

***Scheme B*** We randomly select 500 case families (number of cases greater than or equal to 1 per family) and 500 control families (0 cases per family) from the simulated foundation data. (*K*=0.808)

***Scheme C*** We select 5000 cases by including all cases from case families and randomly select 5000 controls from all control families based on the simulated foundation data. (*K*=1)

### Empirical FDR and power

We applied GhostKnockoffs and the second-order knockoffs to the simulation data. We ran 1000 replicates and estimated empirical FDR and power. The empirical FDR is estimated by the proportion of false positive variants among all selected ones. The empirical power is estimated by the proportion of selected variants among all causal ones.

### Meta-analysis Z-scores accounting for sample overlapping

Consider *L* studies with overlapping samples, the *l^th^* study has sample size *n_l_* and Z-scores ***Z_l,score_***. He et al.^9^ propose a weighting scheme that accounts for sample overlapping and maximizes the power of meta-analysis. The optimal weights *w_l_* are the solutions of the following optimization problem:

$$\text{minimize}\sum_{1\leq i,j\leq L}w_{i}w_{j}Cor.S_{ij},\text{subject to}\sum_{l}w_{l}\sqrt{n_{l}}=1,w_{l}\geq 0,$$

where *Cor. S_ij_* denotes study correlations caused by sample overlapping and is calculated using corresponding Z-scores and LD. We then define meta-analysis Z-score as:

$$Z=H\underset{l}{\sum }w_{l}\text{*}C_{l}Z_{l},$$

***C_l_*** = *diag*(*c*_*l*1_, …, *c_lp_*) is a diagonal matrix where *c_lj_* = 1 if *Z_lj_* is observed and *c_lj_* = 0 otherwise; ***H*** = *diag*(*h*_1_, …, *h_p_*) where $h_{j}=\frac{1}{\sqrt{\sum_{1\leq s,t\leq L}w_{s}w_{t}c_{sj}c_{tj}\text{cor}.S_{st}}}$.

Similar methods have been proposed by Lin and Sullivan^23^ and implemented in METAL (https://genome.sph.umich.edu/wiki/METAL_Documentation). The calculated Z-scores are used as the input of the Ghostknockoffs inference. We demonstrate valid FDR control of using meta-analysis Z-scores as input in our simulation studies. This significantly simplified the meta-analysis pipeline proposed by He et al.^9^ because one no longer needs to perform knockoffs inference for each study separately.

## Results

### Simulation results

Our simulation studies aim to evaluate the robustness of GhostKnockoffs inference to various types of input Z-scores in terms of FDR and power, including: (1) Z-scores from generalized linear mixed models that account for sample relatedness; (2) Z-scores from a meta-analysis of multiple studies; (3) Z-scores from statistical tests that are deviated from usual score tests. To mimic the real data scenario, we directly simulate genetic data of unrelated individuals based on the haplotype dataset from the R package *SKAT*^21^, which mimics the LD structure of the European ancestry. For simulation studies that involve sample relatedness, we additionally simulate offspring genotypes using the gene dropping algorithm^24^ to generate three-generation pedigrees (Figure S11). We then simulate quantitative and dichotomous phenotypes based on generalized linear model for unrelated samples and generalized linear mixed model (with different values of the variance component parameter *θ*) for simulation studies that involve sample relatedness. We present the simulation details in the “Methods” section.

**Figure 2:**
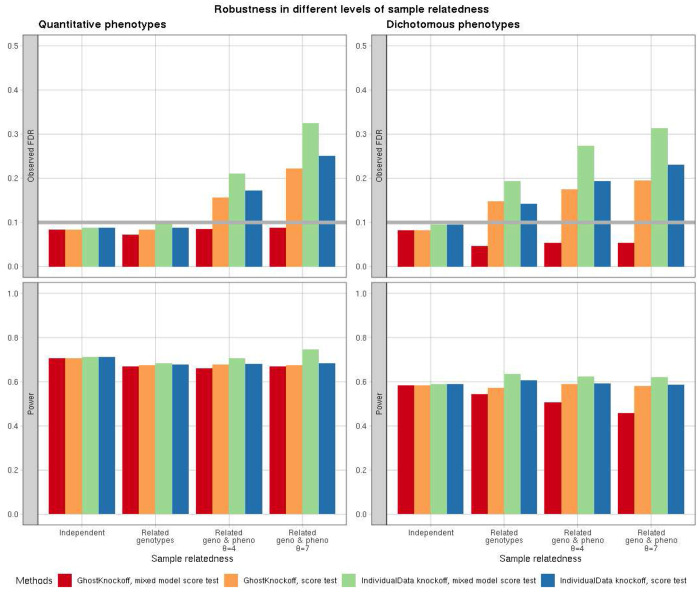
Simulation studies of GhostKnockoffs for different levels of sample relatedness at FDR level 0.1. We present FDR and power estimation of four genotype/phenotype settings of knockoffs tests: GhostKnockoffs and the second-order knockoffs. In the legends, “GhostKnockoff” refers to knockoffs inference based on summary statistics; “IndividualData” refers to knockoffs inference based on generating individual-level knockoffs counterparts; “mixed model score test” refers to score test that adjusts for kinship among phenotypes; “score test” refers to no adjustment for kinship. Each simulated dataset consists of 10,000 samples; FDR (top row) and power (bottom row) are estimated based on 1000 simulations of quantitative (left) and dichotomous (right) phenotypes. Four genotype/phenotype settings are: unrelated genotypes/phenotypes (denoted as “Independent”), related genotypes simulated from the three-generation pedigree and related phenotypes simulated from mixed effect model with variance component parameter *θ* indicating the sample relatedness level.

For (1), we compare GhostKnockoffs with the second-order knockoffs that generate individual-level knockoffs per sample without the consideration of relatedness structure, referred to as “IndividualData knockoffs” in figures of the simulation results. The feature importance scores of two methods are set to be the same, square of Z-scores based on marginal association tests, to achieve unbiased comparison. We considered two types of marginal association tests: score test that ignores sample relatedness and score test from a generalized linear mixed model that correctly accounts for sample relatedness among the phenotypes. In Figure S1, we present QQ-plots of the two tests for each scenario, which confirm the validity of the simulation setting. We present the simulation results in Figure 2.

We observe that for unrelated individuals, all methods control FDR and exhibit similar power. This confirms their theoretical equivalence in this scenario as shown by He et al.^9^. For scenarios where the different individuals share correlated genotypes due to family relatedness, the FDR inflation starts to appear. GhostKnockoffs inference with input Z-scores from generalized linear mixed model is the only method that has valid FDR control. The FDR inflation of the other three methods is more pronounced with higher level of sample relatedness and for scenarios in which genotypes and phenotypes are both related. The empirical results demonstrate that GhostKnockoffs, paired with commonly used generalized linear mixed model score test, can be naturally applied to genetic studies with sample relatedness to perform conditional independent tests with valid FDR control. We present additional comparisons at other FDR levels and other levels of sample relatedness in Figure S2–S5.

**Figure 3:**
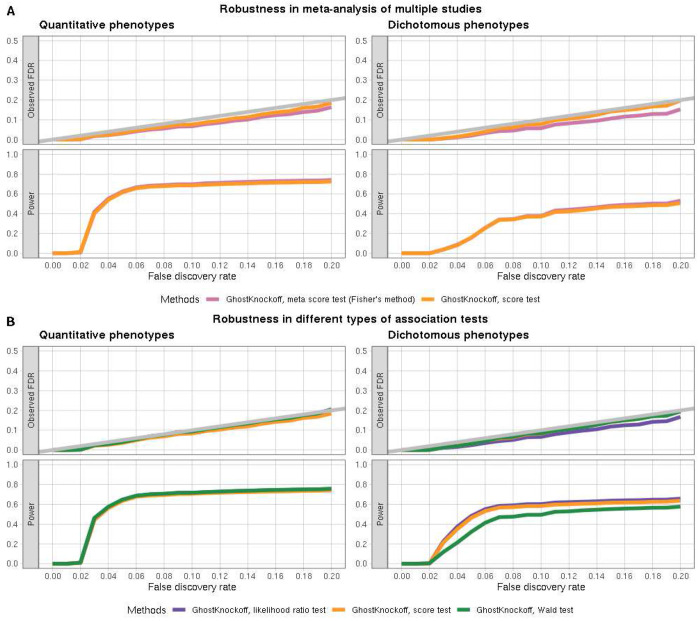
Simulation studies of GhostKnockoffs for meta-analysis of multiple studies and different types of association tests. **A.** FDR and power estimation of GhostKnockoffs with input Z-scores from: (1) meta-analysis of two independent studies; (2) pooled analysis of individual data from two studies. In the legends, “GhostKnockoff” refers to knockoffs inference based on summary statistics; “score test” refers to score test applied to samples pooled from the two independent studies; “meta score test (Fisher’s method):” refers to a meta-analysis of score test p-values calculated separately from two independent studies using Fisher’s method. Each simulated dataset consists of 10,000 independent samples; FDR (top row) and power (bottom row) are estimated based on 1000 simulations of quantitative (left) and dichotomous (right) phenotypes. **B.** FDR and power estimation of GhostKnockoffs with input Z-scores from different types of association tests. In the legends, “GhostKnockoff” refers to knockoffs inference based on summary statistics; “likelihood ratio test” refers to likelihood ratio test applied to independent samples; “score test” refers to score test applied to independent samples; “Wald test” refers to Wald test applied to independent samples. Each simulated dataset consists of 10,000 independent samples; FDR (top row) and power (bottom row) are estimated based on 1000 simulations of quantitative (left) and dichotomous (right) phenotypes.

For (2), we compare GhostKnockoffs with input Z-scores from: a. meta-analysis of two independent studies (e.g. using the Fisher’s combined probability test); b. pooled analysis of individual data from two studies. We present the simulation results in Figure 3A. We observed that the two methods exhibit nearly equivalent FDR and power. The results demonstrate that GhostKnockoffs based on proper meta-analysis of Z-scores guarantee rigorous FDR control and demonstrate similarly high power.

For (3), we compare GhostKnockoffs based on Z-scores from different types of association tests (score test, Wald test and likelihood ratio test). We present simulation results in Figure 3B. We observed that all three methods controlled FDR and exhibited similar power. GhostKnockoffs demonstrate robustness towards different association tests that yield Z-scores as the measurement of single-variant feature importance.

### Meta-analysis of Alzheimer’s disease (AD) studies

We applied GhostKnockoffs to the meta-analysis of Z-scores that aggregates nine European ancestral GWASs and whole exome/genome sequencing (WES/WGS) studies to select putative causal variants for AD. The calculation of the meta-analysis of Z-scores accounting for sample overlapping can be found in the “Method” section. The nine studies include: (1) a genome-wide survival analysis of AD samples from the International Genomics of Alzheimer’s Project (IGAP) (14,406 cases and 25,849 controls)^25^; (2) a meta-analysis of GWASs in AD and AD-by-proxy based on parental diagnosis (71,880 cases and 383,378 controls)^26^; (3) a meta-analysis of GWASs in clinically diagnosed late-onset AD samples from the IGAP (21,982 cases and 41,944 controls)^27^; (4) a meta-analysis of GWASs and GWASs-by-proxy in AD (53,042 cases and 355,900 controls)^28^; (5) GWASs of 32 AD cohorts (65,701 participants)^29^; (6-7) WES analyses of the Alzheimer’s Disease Sequencing Project (ADSP) by Bis et al. (5,740 cases and 5,096 controls)^30^ and Le Guen et al. (6,008 cases and 5,119 controls)^29^; (8-9) exome/genome-wide association analyses of ADSP (6,155 cases and 5,418 controls for ADSP WES, 3,584 cases and 2,949 controls for ADSP WGS)^31^. Three^26–28^ out of the nine AD GWASs and whole exome/genome sequencing studies incorporate data from the UK Biobank. Therefore, the meta-dataset reflects a relevant level of sample relatedness. The meta-summary-level statistics consist of 9,195,254 common and low minor-allele-frequency (MAF) genetic variants with MAF>0.01.

The genetic variants are later matched with the European LD reference panel from the gnomAD by chromosome, base pair position and reference/alternative alleles. We exclude variants if: (1) they do not show up in the gnomAD; (2) they do not pass the gnomAD quality control; (3) they are multi-allelic; (4) they are in low complexity regions. To improve the power of conditional independent tests in presence of tightly correlated variants, we applied a single linkage hierarchical clustering with a correlation cutoff of 0.75 and used one representative variant per cluster for GhostKnockoffs inference. At FDR levels 0.05/0.1/0.2, we report the selected representative variants together with their neighboring variants in the same cluster which are (1) in high LD (0.75 or more correlated with the representative ones) and (2) of significant signal strength (absolute values of marginal Z-scores no less than those of corresponding selected representative ones) as the variable selection results. To pinpoint the mechanism-based gene that is most likely linked to an identified variant, we applied the Open Targets V2G strategy. We also reported other linking strategies, such as the cS2G and proximal genes (nearest genes) in the “Supplemental Materials”.

**Figure 4:**
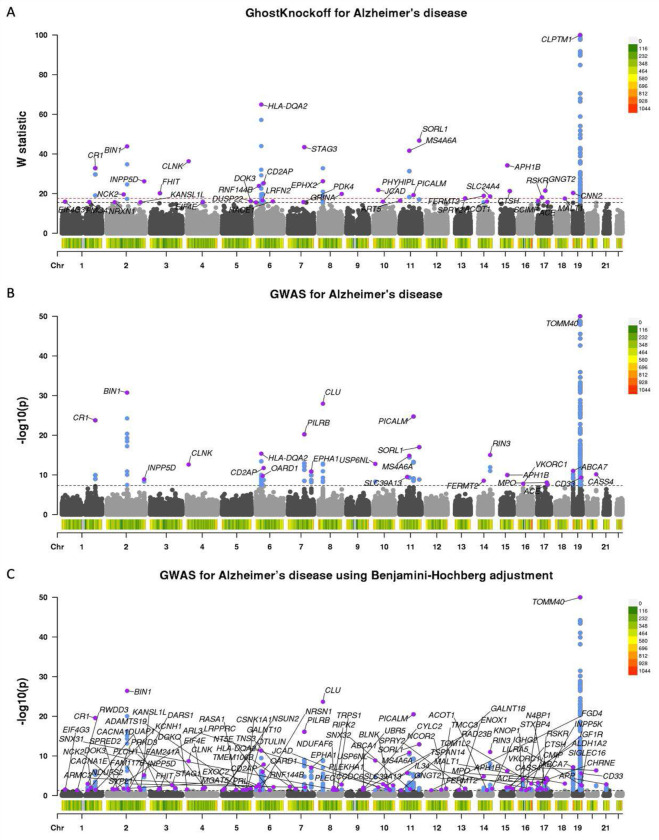
Meta-analysis of Alzheimer’s disease studies. We present Manhattan plots of GhostKnockoffs and conventional GWASs applied to the meta-analysis of Z-scores that aggregate nine European ancestral GWASs and WES/WGS studies. Each locus is annotated with the V2G gene corresponding to the variant with the minimum p-value. The variant density of each independent locus (number of variants per 1Mb) is shown at the bottom of plots. **A.** Manhattan plot of *W* statistics (truncated at 100) based on GhostKnockoffs at FDR levels 0.05 (red horizontal dashed line) and 0.1 (black horizontal dashed line). **B.** Manhattan plot of −log_10_(p-value) (truncated at 50) based on conventional GWASs at p-value threshold 5 × 10^−8^ (black horizontal dashed line). **C.** Manhattan plot of −log_10_(Benjamini–Hochberg adjusted GWASs p-value) (truncated at 50) at threshold 0.05 (black horizontal dashed line).

We present GhostKnockoffs results in Figure 4A, conventional GWASs results (at p-value threshold 5 × 10^−8^) in Figure 4B, and Benjamini–Hochberg adjusted GWASs results (at threshold 0.05) in Figure 4C. The performance of GhostKnockoffs is compared with GWASs in terms of discoveries of independent loci, which are defined as loci that are at least 1Mb away from one another. The Manhattan plots present the identified independent loci, and each locus is annotated with the Open Targets Genetics V2G gene corresponding to the variant with the minimum p-value. In Figure S6A–B, we present Manhattan plots based on a different linking strategy, where each locus is annotated with the cS2G gene that appears most frequently.

**Figure 5:**
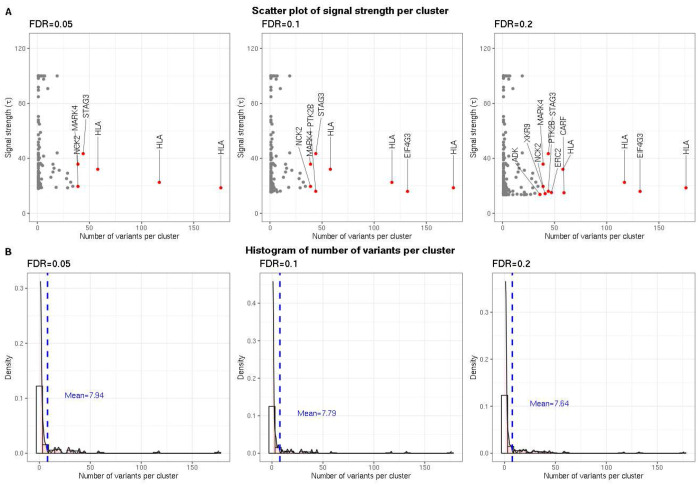
Genetic variants of hierarchical clusters selected by GhostKnockoffs. **A.** Scatter plots of clusters selected by GhostKnockoffs at FDR levels 0.05/0.1/0.2. The x-axis denotes the number of variants of each selected cluster. The y-axis denotes the signal strength (*τ* denotes the difference between the highest importance score, measured by the square of Z-scores, and the median of remaining importance scores) of the selected representative variant of each cluster. The signal strength *τ* is truncated at 100 for better visualization. Red points denote clusters consisting of no less than 35 variants with corresponding gene region annotated above. **B.** Histograms of the number of variants per cluster selected by GhostKnockoffs at FDR levels 0.05/0.1/0.2. The blue vertical dashed line denotes the average number of variants per cluster.

We observed that GhostKnockoffs identified 25 risk loci at FDR=0.05, 41 loci at FDR=0.1, and 74 at FDR=0.2. GWASs identified 25 loci at p-value threshold 5 × 10^−8^. GWASs with Benjamini–Hochberg adjustment identified 103 loci at threshold 0.05. GhostKnockoffs adopt the FDR control to identify small effect risk loci that currently lie below the genome-wide significance threshold even in large GWASs. With more selected significant loci, GhostKnockoffs identify weak associations missed by GWASs that may explain additional AD phenotypic variance. Overall, our meta-analysis highlights two important distinctions between GhostKnockoffs and GWASs. First, GhostKnockoffs control FDR, a more liberal but still statistically rigorous criterion, whereas GWASs control FWER using empirical p-value threshold 5 × 10^−8^. Second, GhostKnockoffs perform a conditional association test, whereas GWASs rely on marginal association testing. We anticipate that the more liberal FDR control leads to identification of more associated loci, while the conditional association testing approach should reduce false positive findings caused by LD confounding at each locus. We observed that the median number of clusters selected by GhostKnockoffs per locus was 1. Each cluster included neighboring variants (1) in high LD with the representative one and (2) with relatively strong marginal genotype-phenotype associations. We noticed that there existed loci with variants in high LD (e.g., HLA) where GhostKnockoffs failed to distinguish the putative causal variants from the proxy variants. GhostKnockoffs tended to select a large cluster in such scenarios (Figure 5).

As GhostKnockoffs may fail to distinguish the putative causal variants from the proxy variants in high LD regions, we performed sensitivity analysis after removing these regions (HLA region located at chr6:28,510,120-33,480,577; CARF region located at chr2:202,912,214-202,988,263; and EIF4G3 region located at chr1:20,806,292-21,177,285 according to the Genome Reference Consortium of the National Institutes of Health National Center for Biotechnology Information). Figure S7A–B presents Manhattan plots comparing the results from GhostKnockoffs, which identified 27 loci at FDR=0.1, and GWASs, which identified 19 loci with p-value threshold 5 × 10^−8^.

We leveraged the Open Targets Genetics V2G strategy to map genetic variants selected by GhostKnockoffs to functional genes. Table S1 summarizes the 41 independent loci associated with AD based on GhostKnockoffs at FDR=0.1, including variants’ basic information, GhostKnockoffs statistics, and disease susceptibility genes identified by the V2G and cS2G strategies, proximal genes, and other enrichment analysis results.

### Enrichment analysis of single-cell transcriptomics data

We validated functional genes implicated by GhostKnockoffs using the differentially expressed gene (DEG) analysis of single-cell RNA sequences (scRNAseq) consisting of 143,793 single-nucleus transcriptomes from two human brain components, hippocampus (9 AD cases 8 controls) and cortex (4 AD cases and 4 controls). We performed the DEG analysis at the gene level using R package *Seurat*, stratified by 14 cell types (veinous endothelial cell, T cell, smooth muscle cell, pericyte, capillary endothelial cell, arterial endothelial cell, oligodendrocyte, perivascular fibroblast, ependymal cell, microglia, astrocyte, oligodendrocyte progenitor cell, meningeal fibroblast, neuron), adjusting for age, batch effect, cellular detection rate and relatedness within samples.

**Figure 6:**
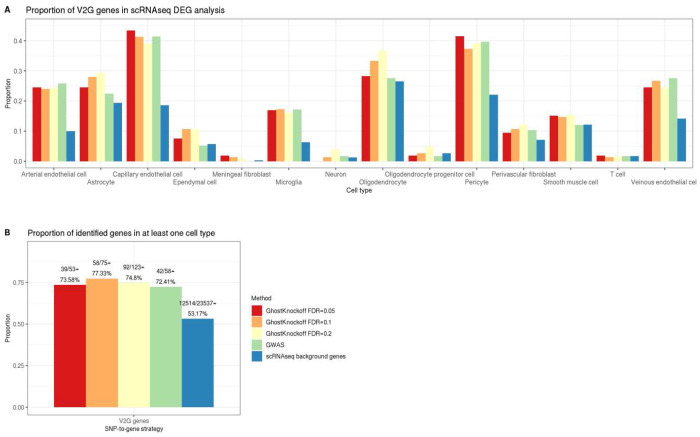
Enrichment analysis of single-cell transcriptomics data for identified AD-related genes. We present differentially expressed gene (DEG) analysis of single-cell RNA sequences consisting of 143,793 single-nucleus transcriptomes for AD-related genes identified by the cS2G strategy based on GhostKnockoffs’ and conventional GWASs’ variable selection results to validate their signal enrichment. **A.** We present proportions of DEGs identified by the V2G strategy stratified by 14 cell types. Genes are classified as DEGs if their DE analysis raw p-values are smaller than 0.05. **B.** We present proportions of DEGs identified by the V2G strategy based on GhostKnockoffs (FDR=0.05/0.1/0.2), GWASs and all background genes. Genes are classified as DEGs if any of the 14 cell types of DE analysis raw p-values is smaller than 0.05.

We present the scRNAseq DEG analysis results of V2G genes based on GhostKnockoffs at FDR levels 0.05/0.1/0.2 and conventional GWASs in Figure S8A–D. The volcano plots demonstrate genes’ differential expression among 14 cell types in terms of (1) log_2_(fold change) of average expression between AD cases and controls, (2) −log_10_ (p-value) of DE testing. Two thresholds, 0.05 and Bonferroni adjustment 0.05/(number of genes with expression measurements), are used to determine DEGs. Figure 6A and Figure S9A–B compare proportions of differentially expressed V2G, cS2G, and proximal genes based on GhostKnockoffs at FDR levels 0.05/0.1/0.2 and GWASs. We could see that GhostKnockoffs and GWASs enrich AD-related genes since their proportions of DEGs are constantly higher than the baseline, which is defined as the proportion of DEGs among all 23,537 background genes from the scRNAseq data, across all cell types. Figure 6B and Figure S9C shows that proportions of differentially expressed V2G, cS2G, and proximal genes in at least one cell type based on GhostKnockoffs and conventional GWASs are generally higher than the baseline. At FDR level 0.1, 58/75=77.33% V2G genes (or 53/73=72.6% proximal genes, 55/79=69.62% cS2G genes) based on GhostKnockoffs variable selection results are differentially expressed in at least one cell type and are significantly higher than the baseline 12,514/23,537=53.17%. Table S2–S4 show detailed counts and percentages of differentially expressed genes identified by V2G, cS2G strategies and proximal genes based on GhostKnockoffs’ and conventional GWASs’ variable selection results. We see that GhostKnockoffs capture more weak associations of AD-related variants, whose disease susceptibility genes identified by the V2G, cS2G strategies or proximal genes are of higher magnitude compared to the baseline. The functional analysis shows that these additional genes, despite being weaker signals, are still highly enriched compared to other background genes. Interestingly, both methods demonstrate similar proportions of DEG enrichment in the scRNAseq analysis.

We also implemented a locus-level ranking-based approach to assess the biological enrichment of putative causal variants identified by GhostKnockoffs compared to those identified by GWASs. First, we compiled all linked genes within the 25 independent loci identified by GWASs, creating a gene set referred to as *Genes_GWAS*. Next, we identified the top 25 loci using GhostKnockoffs, with ranking based on the W-statistics, and summarized all linked genes within these loci as the *Genes_GhostKnockoffs* set. We then performed a rank-based enrichment analysis for both gene sets, *Genes_GWAS* and *Genes_GhostKnockoffs*. Figure S10A–C compare the proportions of differentially expressed V2G, cS2G, and proximal genes at the top 25 independent loci identified by GhostKnockoffs and GWASs. The results indicate that both GhostKnockoffs and GWASs effectively enrich for AD-related genes, as their proportions of differentially expressed genes are generally higher than the baseline across most cell types. Additionally, Figure S10D demonstrates that the proportions of differentially expressed V2G, cS2G, and proximal genes in at least one cell type, as identified by GhostKnockoffs and GWASs, are also generally higher than the baseline.

## Discussion

We have proposed a simple and effective knockoffs-based method, which is valid in the presence of relatedness individuals and requires only summary statistics from conventional GWASs. It provides a solution to the issue of FDR inflation that commonly affects knockoffs inference when sample relatedness is present. We show that the GhostKnockoffs inference is robust to cryptic relationship or overlap in the underlying study populations, as long as the input summary statistics are from valid marginal association tests and their correlations are consistent with the correlations among the corresponding genetic variants. Our method combines Ghostknockoffs with appropriate “upstream” summary statistics from GWASs and adequately controls FDR, while vanilla knockoffs with individual-level data can be inflated. These results generalize the application of GhostKnockoffs to broader GWASs settings, such as the meta-analysis of multiple overlapping studies and studies based on association test statistics deviated from score tests.

We applied GhostKnockoffs to the meta-analysis of nine European ancestral GWASs and WES/WGS studies to select putative causal genetic variants for AD. GhostKnockoffs identified much more loci that were missed by conventional GWASs. We also leveraged and compared external SNPs-to-gene strategies such as the Open Targets V2G pipeline to further pinpoint functionally informed genes for the selected variants. Finally, we performed downstream analysis of transcriptomics data to validate the enrichment of biological functionality of the mapped genes. The meta-analysis of AD studies demonstrate that GhostKnockoffs improve the power to identify putative causal variants with weak genotype-phenotype associations and mechanism-based genes.

One limitation of GhostKnockoffs is that there are genetic regions with variants in high LD and GhostKnockoffs fail to distinguish the putative causal variants from proxy ones. Since proxy variants in high LD decrease the power to identify causal ones, variants are then grouped into hierarchical clusters first and GhostKnockoffs are applied to select the representative variant from each cluster. In this case, even though theoretically, GhostKnockoffs adjust for the LD by preserving the correlation structure of genotypes among knockoffs counterparts, it fails to identify putative causal variants within the same hierarchical cluster. To avoid missing the “real” causal variants, the selected representative variants and neighboring ones from the same cluster are treated as GhostKnockoffs’ variable selection results, meaning that many proxy variants still await a further step of fine-mapping. We are interested in exploring alternative knockoffs inference methods to integrate biological annotations and other supportive information as the side information for the original variants to further fine-map putative causal variants from proxy ones.

## Supplementary Material

1

## Data Availability

Summary statistics utilized in this manuscript were derived from existing studies available through the UK Biobank (https://pheweb.org/UKB-SAIGE/). Specific summary statistics for genome-wide association studies (GWAS) on Alzheimer’s disease are accessible via several repositories: Huang et al., 2017 (NIAGADS ID: NG00058), Jansen et al., 2019 (available at CTG Lab), Kunkle et al., 2019 (NIAGADS ID: NG00075), Schwartzentruber et al., 2021 (GWAS catalog ID: GCST90012877) GWAS Catalog, In-house genome-wide association study imputed using TOPMed reference panels, Bis et al., 2020 (NIAGADS ID: NG00065). Le Guen et al., 2021 (NIAGADS ID: NG000112). In-house whole-exome sequencing analysis of ADSP (NIAGADS ID: NG00067.v5), In-house whole-genome sequencing analysis of ADSP (NIAGADS ID: NG00067.v5). Additionally, the single-cell RNA-Seq data for the candidate genes can be accessed via the GEO database under accession code GSE163577. We have implemented the proposed pipeline in R that can be accessed at https://github.com/xinranqi/RobustInferenceWithGhostKnockoffs.
