# Supplementary material for: Robust inference with GhostKnockoffs in genome-wide association studies
Source: Res Sq. 2025 May 5:rs.3.rs-6396196. Preprint. [Version 1] doi: 10.21203/rs.3.rs-6396196/v1 (PMC12083671; doi:10.21203/rs.3.rs-6396196/v1)
Supplement: 1 [file NIHPPrs6396196v1-supplement-1.pdf]

Supplemental Figures

Figure S1: QQ plots of simulation studies. We present QQ plots of simulation studies based on 1000 replicates of 10,000 samples for quantitative (left column 1) and dichotomous (right columns 2-3) phenotypes. The left column panel shows QQ plots for independent/related genotypes and quantitative phenotypes. The right three right column panels show QQ plots for independent/related genotypes and dichotomous phenotypes simulated from three sampling schemes. **Scheme A.** We randomly select 5000 cases and 5000 controls from the simulated foundation data. **Scheme B.** We randomly select 500 case families (number of cases greater than or equal to 1 per family) and 500 control families (0 cases per family) from the simulated foundation data. **Scheme C**. We select 5000 cases by including all cases from case families and randomly select 5000 controls from all control families based on the simulated foundation data. The genomic inflation factors, denoted as "lambda gc", of two association tests (score test and mixed model score test) are listed in the upper-left corner of each QQ plot. The divergence between score test and mixed model score test demonstrates the phenotype relatedness due to (1) random effects of mixed effect model to simulate phenotypes, (2) additional relatedness caused by sampling schemes.

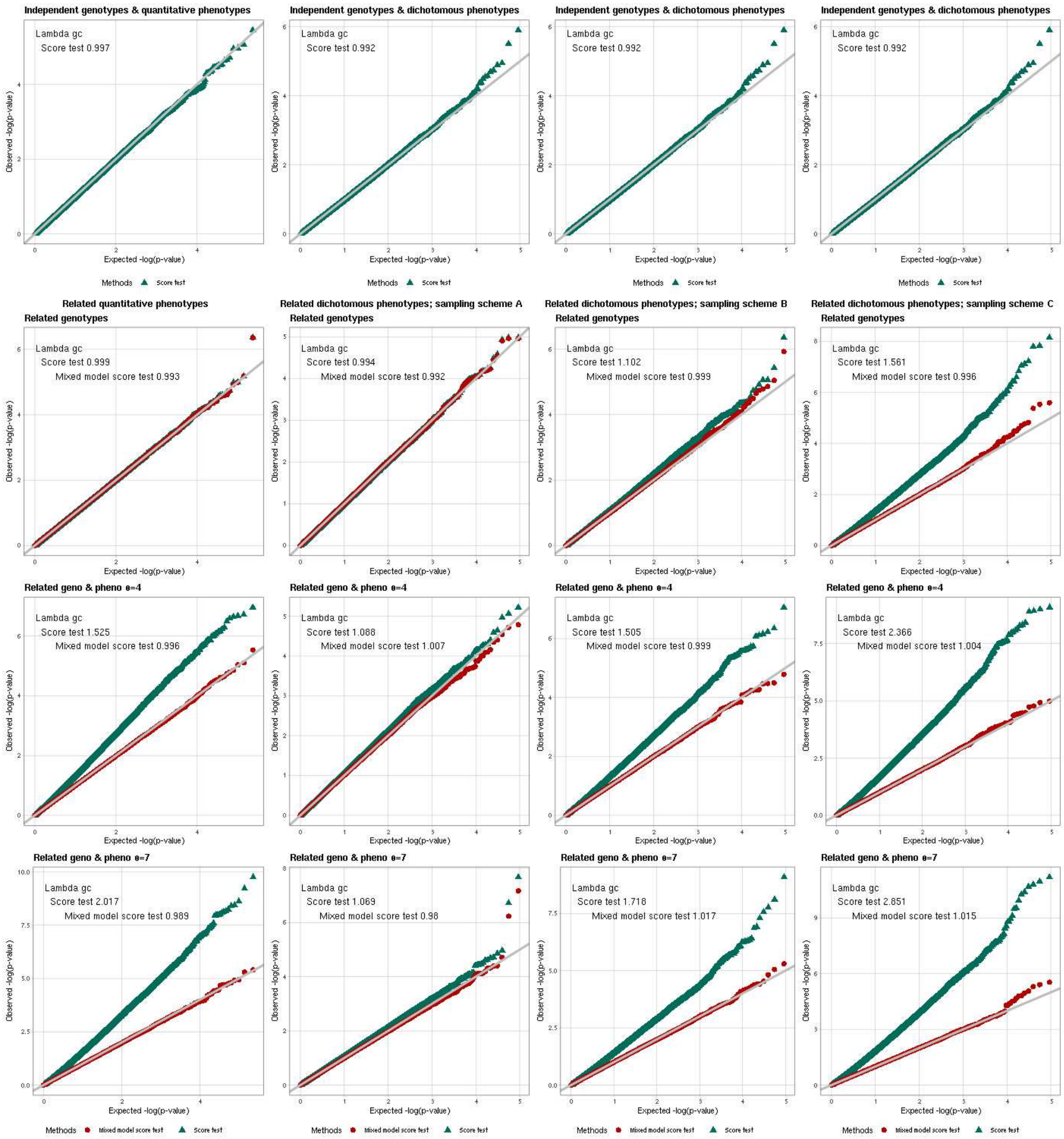
Independent genotypes & quantitative phenotypes
Lambda gc
Score test 0.997
Independent genotypes & dichotomous phenotypes
Lambda gc
Score test 0.992
Independent genotypes & dichotomous phenotypes
Lambda gc
Score test 0.992
Independent genotypes & dichotomous phenotypes
Lambda gc
Score test 0.992
Related quantitative phenotypes
Related genotypes
Lambda gc
Score test 0.999
Mixed model score test 0.993
Related dichotomous phenotypes; sampling scheme A
Related genotypes
Lambda gc
Score test 0.994
Mixed model score test 0.992
Related dichotomous phenotypes; sampling scheme B
Related genotypes
Lambda gc
Score test 1.102
Mixed model score test 0.999
Related dichotomous phenotypes; sampling scheme C
Related genotypes
Lambda gc
Score test 1.561
Mixed model score test 0.996
Related geno & pheno θ=4
Lambda gc
Score test 1.525
Mixed model score test 0.996
Related geno & pheno θ=4
Lambda gc
Score test 1.088
Mixed model score test 1.007
Related geno & pheno θ=4
Lambda gc
Score test 1.505
Mixed model score test 0.999
Related geno & pheno θ=4
Lambda gc
Score test 2.366
Mixed model score test 1.004
Related geno & pheno θ=7
Lambda gc
Score test 2.017
Mixed model score test 0.989
Related geno & pheno θ=7
Lambda gc
Score test 1.069
Mixed model score test 0.98
Related geno & pheno θ=7
Lambda gc
Score test 1.718
Mixed model score test 1.017
Related geno & pheno θ=7
Lambda gc
Score test 2.851
Mixed model score test 1.015
Observed -log(p-value)
Expected -log(p-value)
Methods
Mixed model score test
Score test

Figure S2: Simulation studies of GhostKnockoffs for different levels of sample relatedness at FDR level 0.1. We present FDR and power estimation of four genotype/phenotype settings of knockoffs tests: GhostKnockoffs and the second-order knockoffs. In the legends, "GhostKnockoff" refers to knockoffs inference based on summary statistics; "IndividualData" refers to knockoffs inference based on generating individual-level knockoffs counterparts; "mixed model score test" refers to score test that adjusts for kinship among phenotypes; "score test" refers to no adjustment for kinship. Each simulated dataset consists of 10,000 samples; FDR (top row) and power (bottom row) are estimated based on 1000 simulations of quantitative (upper left) and dichotomous (lower left, right) phenotypes. Four genotype/phenotype settings are: unrelated genotypes/phenotypes (denoted as "Independent"), related genotypes simulated from the three-generation pedigree and related phenotypes simulated from mixed effect model with variance component parameter θ indicating the sample relatedness level. Dichotomous phenotypes are of varying levels of relatedness due to different sampling schemes of case control family studies.

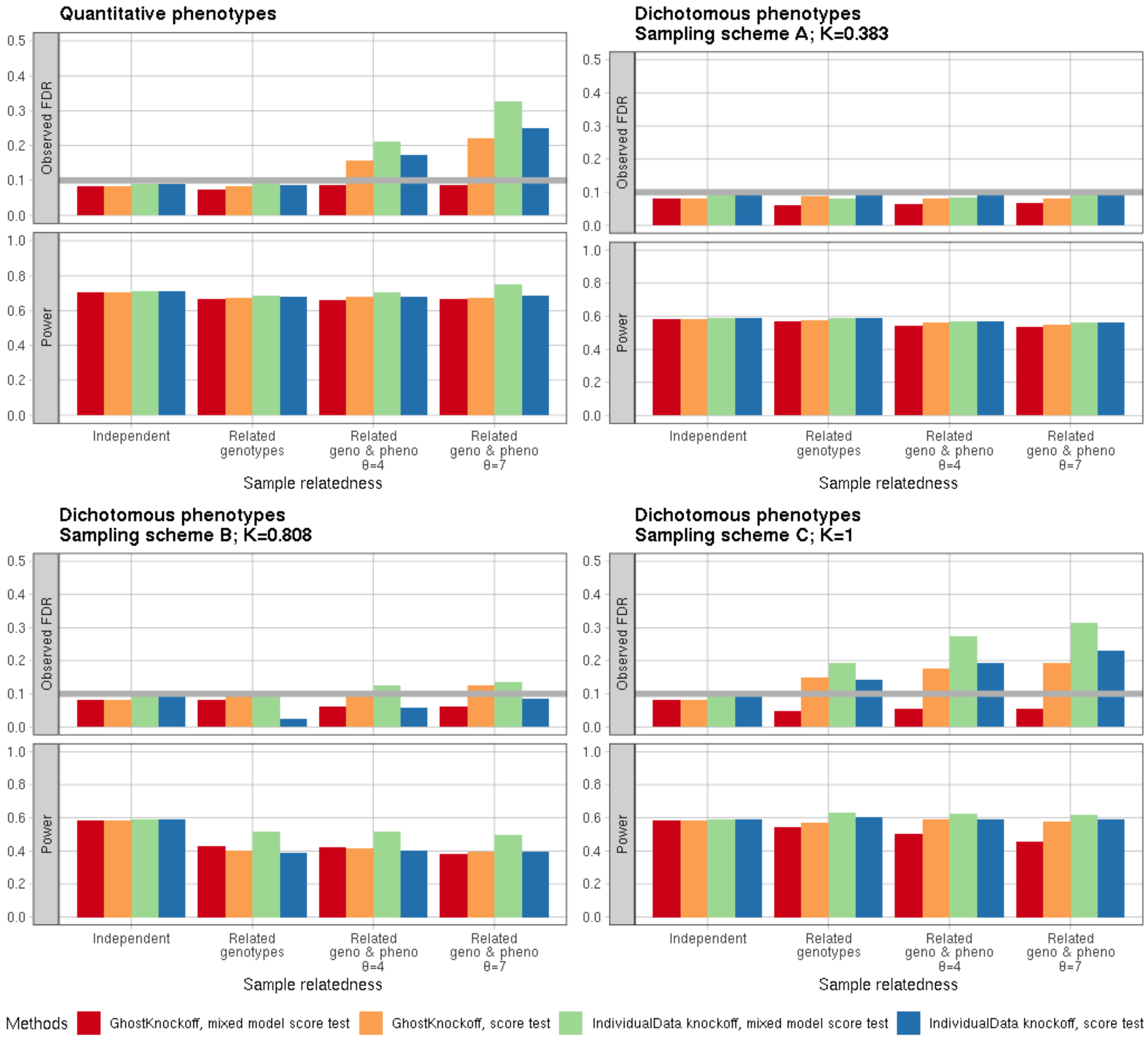
Quantitative phenotypes
Dichotomous phenotypes
Sampling scheme A; K=0.383
Dichotomous phenotypes
Sampling scheme B; K=0.808
Dichotomous phenotypes
Sampling scheme C; K=1
Observed FDR
Power
Independent
Related genotypes
Related geno & pheno θ=4
Related geno & pheno θ=7
Sample relatedness
Methods
GhostKnockoff, mixed model score test
GhostKnockoff, score test
IndividualData knockoff, mixed model score test
IndividualData knockoff, score test

Figure S3: Simulation studies of GhostKnockoffs for different levels of sample relatedness at FDR level 0.2. We present FDR and power estimation of four genotype/phenotype settings of knockoffs tests: GhostKnockoffs and the second-order knockoffs. In the legends, "GhostKnockoff" refers to knockoffs inference based on summary statistics; "IndividualData" refers to knockoffs inference based on generating individual-level knockoffs counterparts; "mixed model score test" refers to score test that adjusts for kinship among phenotypes; "score test" refers to no adjustment for kinship. Each simulated dataset consists of 10,000 samples; FDR (top row) and power (bottom row) are estimated based on 1000 simulations of quantitative (upper left) and dichotomous (lower left, right) phenotypes. Four genotype/phenotype settings are: unrelated genotypes/phenotypes (denoted as "Independent"), related genotypes simulated from the three-generation pedigree and related phenotypes simulated from mixed effect model with variance component parameter θ indicating the sample relatedness level. Dichotomous phenotypes are of varying levels of relatedness due to different sampling schemes of case control family studies.

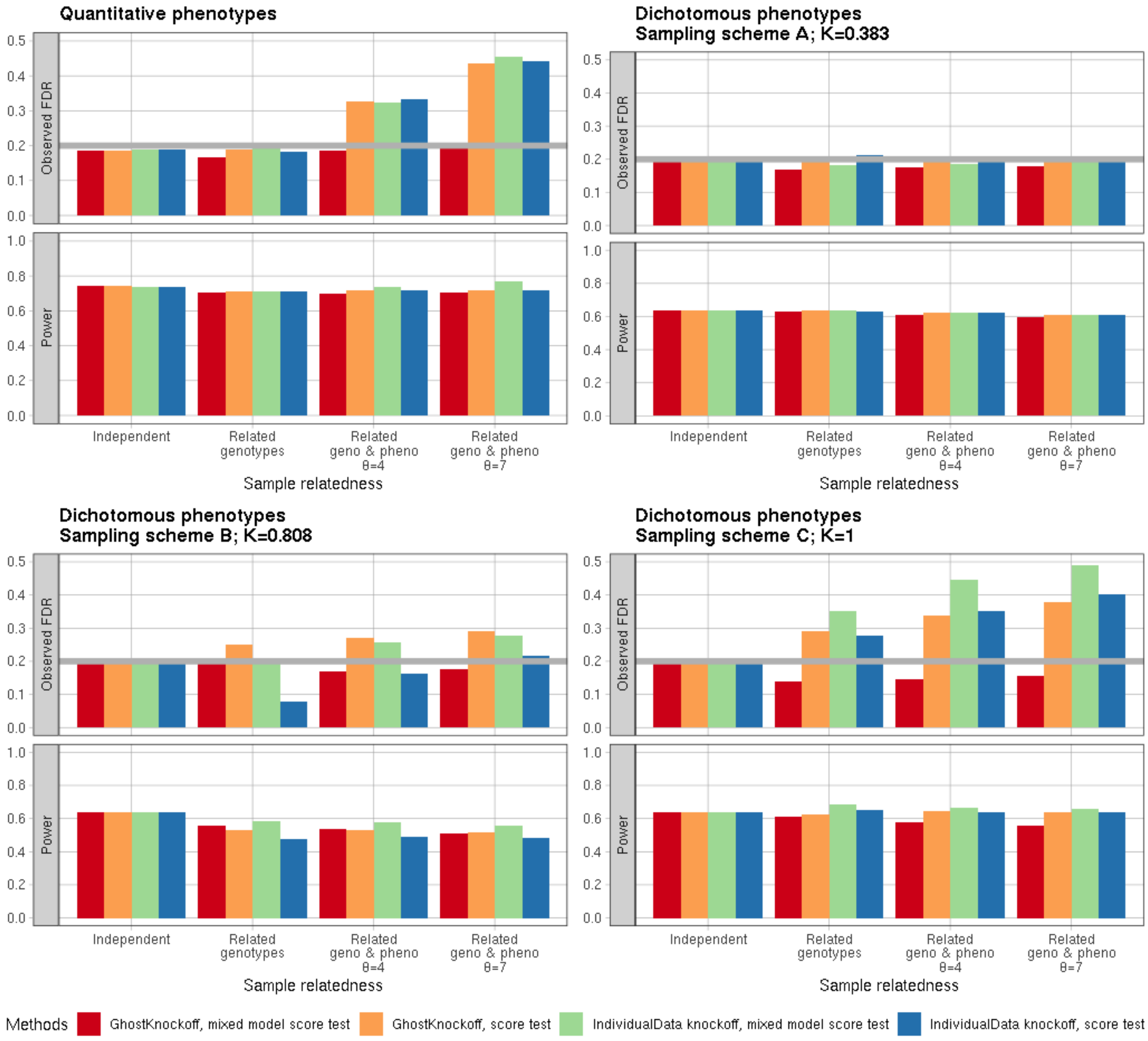

Quantitative phenotypes
Dichotomous phenotypes
Sampling scheme A; K=0.383
Dichotomous phenotypes
Sampling scheme B; K=0.808
Dichotomous phenotypes
Sampling scheme C; K=1
Observed FDR
Power
Independent
Related genotypes
Related geno & pheno θ=4
Related geno & pheno θ=7
Sample relatedness
Methods
GhostKnockoff, mixed model score test
GhostKnockoff, score test
IndividualData knockoff, mixed model score test
IndividualData knockoff, score test

Figure S4: Simulation studies of GhostKnockoffs for quantitative phenotypes. We present FDR and power estimation of four genotype/phenotype settings of knockoffs tests: GhostKnockoffs and the second-order knockoffs. In the legends, "GhostKnockoff" refers to knockoffs inference based on summary statistics; "IndividualData" refers to knockoffs inference based on generating individual-level knockoffs counterparts; "mixed model score test" refers to score test that adjusts for kinship among phenotypes; "score test" refers to no adjustment for kinship. Each simulated dataset consists of 10,000 samples; FDR (top row) and power (bottom row) are estimated based on 1000 simulations of quantitative phenotypes. The top panel shows FDR and power estimates of two methods for unrelated genotypes/phenotypes (denoted as "Independent"). The bottom panels show FDR and power estimates of two methods for related genotypes (simulated using gene dropping algorithm) and phenotypes (simulated from mixed effect model with different values of random effect's variance component parameter θ).

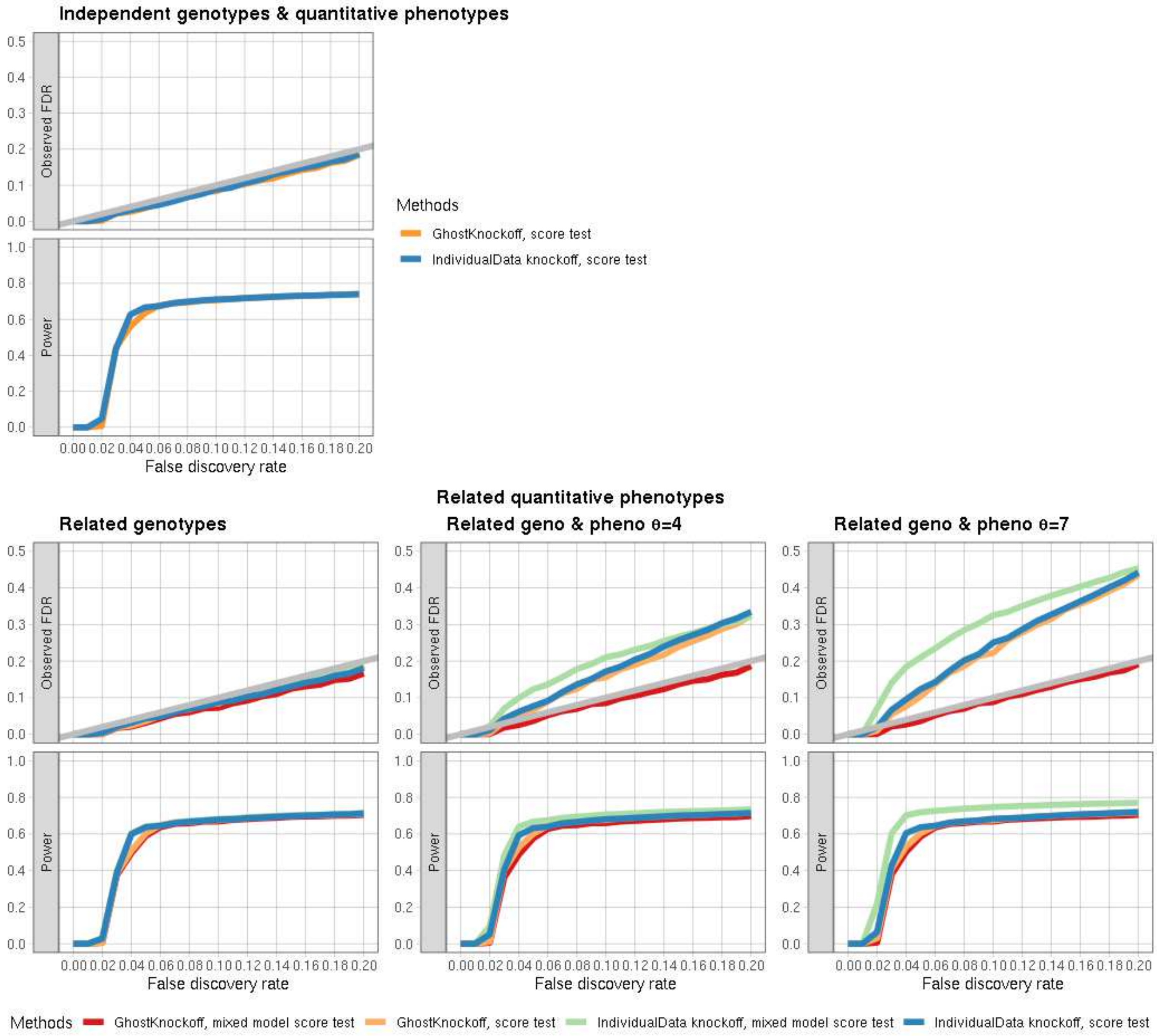

Independent genotypes & quantitative phenotypes
Observed FDR
Power
False discovery rate
Methods
GhostKnockoff, score test
IndividualData knockoff, score test
Related quantitative phenotypes
Related genotypes
Related geno & pheno θ=4
Related geno & pheno θ=7
Methods
GhostKnockoff, mixed model score test
GhostKnockoff, score test
IndividualData knockoff, mixed model score test
IndividualData knockoff, score test

Figure S5: Simulation studies of GhostKnockoffs for dichotomous phenotypes. We present FDR and power estimation of four genotype/phenotype settings of knockoffs tests: GhostKnockoffs and the second-order knockoffs. In the legends, "GhostKnockoff" refers to knockoffs inference based on summary statistics; "IndividualData" refers to knockoffs inference based on generating individual-level knockoffs counterparts; "mixed model score test" refers to score test that adjusts for kinship among phenotypes; "score test" refers to no adjustment for kinship. Each simulated dataset consists of 10,000 samples; FDR (top row) and power (bottom 3 rows) are estimated based on 1000 simulations of dichotomous phenotypes from different sampling schemes of case control family studies. **Scheme A.** We randomly select 5000 cases and 5000 controls from the simulated foundation data. **Scheme B.** We randomly select 500 case families (number of cases greater than or equal to 1 per family) and 500 control families (0 cases per family) from the simulated foundation data. **Scheme C**. We select 5000 cases by including all cases from case families and randomly select 5000 controls from all control families based on the simulated foundation data. The top panel shows FDR and power estimates of two methods for unrelated genotypes/phenotypes (denoted as "Independent"). The bottom 3 panels show FDR and power estimates of two methods for related genotypes (simulated using gene dropping algorithm) and phenotypes (simulated from mixed effect model with different values of random effect's variance component parameter θ).

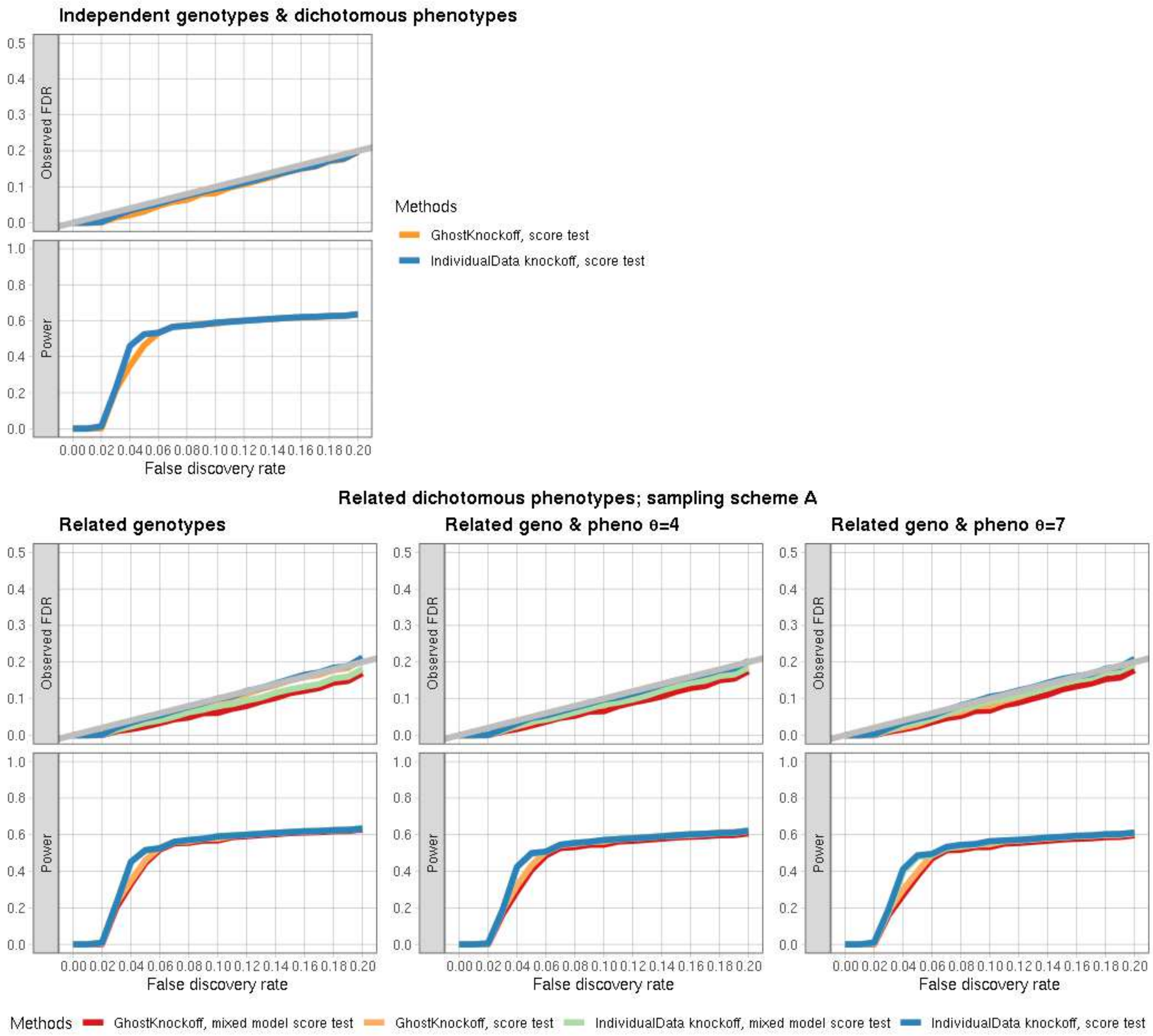
Independent genotypes & dichotomous phenotypes
Observed FDR
Power
False discovery rate
Methods
GhostKnockoff, score test
IndividualData knockoff, score test
Related dichotomous phenotypes; sampling scheme A
Related genotypes
Related geno & pheno θ=4
Related geno & pheno θ=7
Methods
GhostKnockoff, mixed model score test
GhostKnockoff, score test
IndividualData knockoff, mixed model score test
IndividualData knockoff, score test

Figure S5 (continued): Simulation studies of GhostKnockoffs for dichotomous phenotypes.

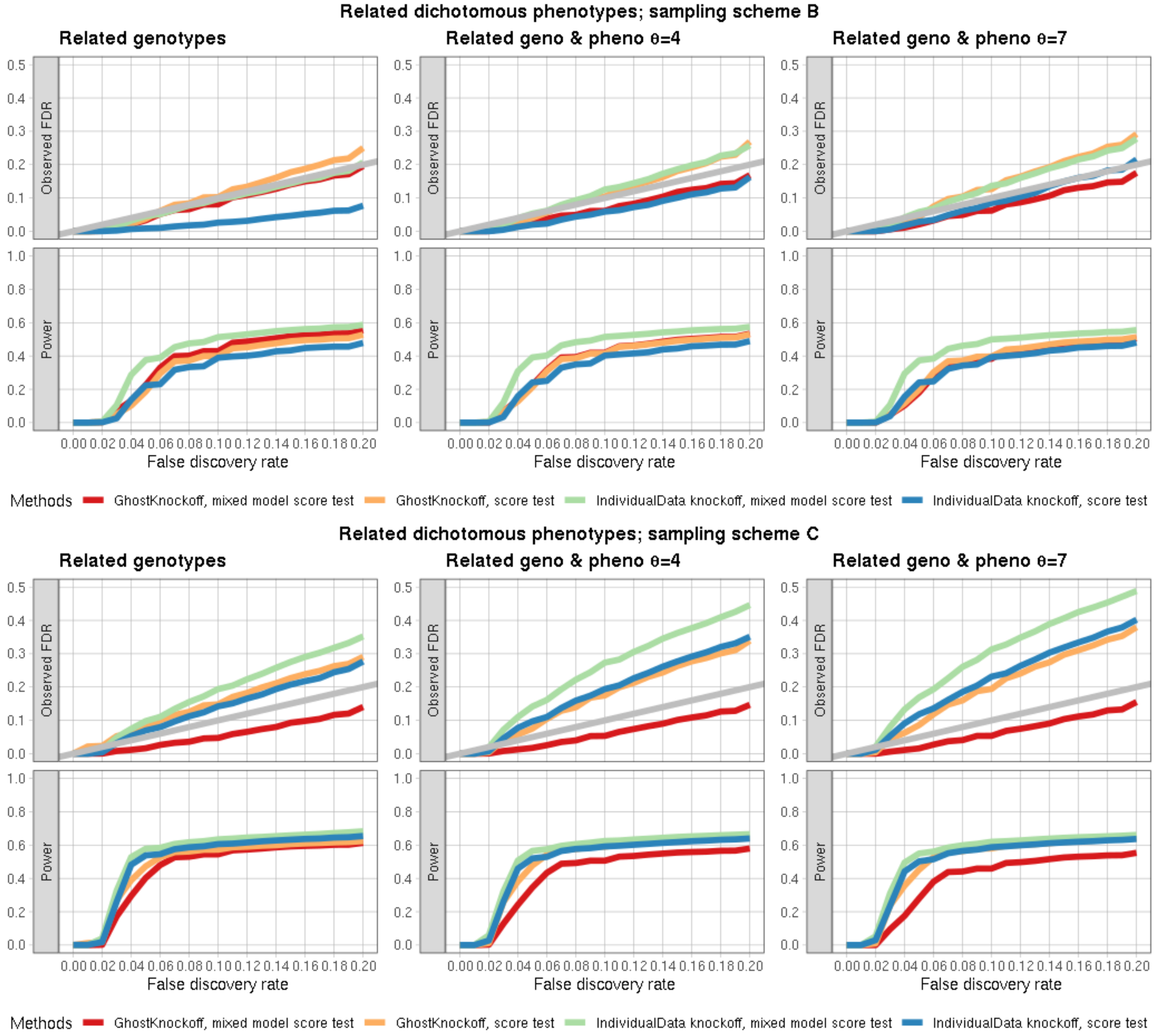

Figure S6: Meta-analysis of Alzheimer's disease studies. We present Manhattan plots of GhostKnockoffs and conventional GWASs applied to the meta-analysis Z-scores that aggregate nine European ancestral GWASs and WES/WGS studies. Each locus is annotated with the cS2G gene that appears most frequently. The variant density of each independent locus (number of variants per 1Mb) is shown at the bottom of plots. **A.** Manhattan plot of W statistics (truncated at 100) based on GhostKnockoffs at FDR levels 0.05 (red horizontal dashed line) and 0.1 (black horizontal dashed line). **B.** Manhattan plot of $-\log_{10}$(p-value) (truncated at 50) based on conventional GWASs at p-value threshold 5×10^{-8} (black horizontal dashed line).

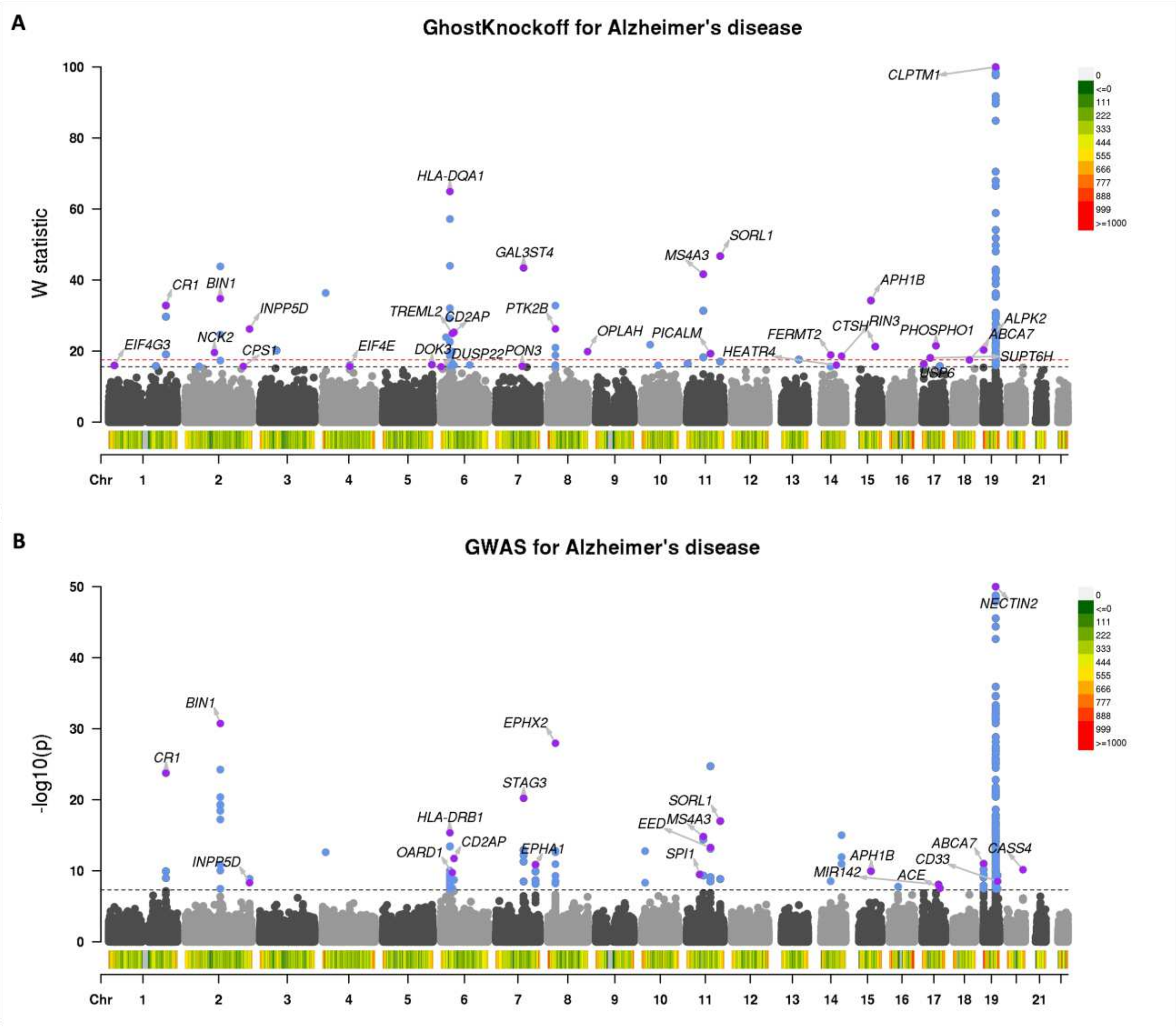

Figure S7: Meta-analysis of Alzheimer's disease studies after removing high LD regions. We present Manhattan plots of GhostKnockoffs and conventional GWASs applied to the meta-analysis of Z-scores that aggregate nine European ancestral GWASs and WES/WGS studies after removing high LD regions (HLA, CARF, and EIF4G3). Each locus is annotated with the cS2G gene corresponding to the variant with the minimum p-value. The variant density of each independent locus (number of variants per 1Mb) is shown at the bottom of plots. **A.** Manhattan plot of W statistics (truncated at 100) based on GhostKnockoffs at FDR levels 0.05 (red horizontal dashed line) and 0.1 (black horizontal dashed line). **B.** Manhattan plot of $-\log_{10}$(p-value) (truncated at 50) based on conventional GWASs at p-value threshold 5×10^{-8} (black horizontal dashed line).

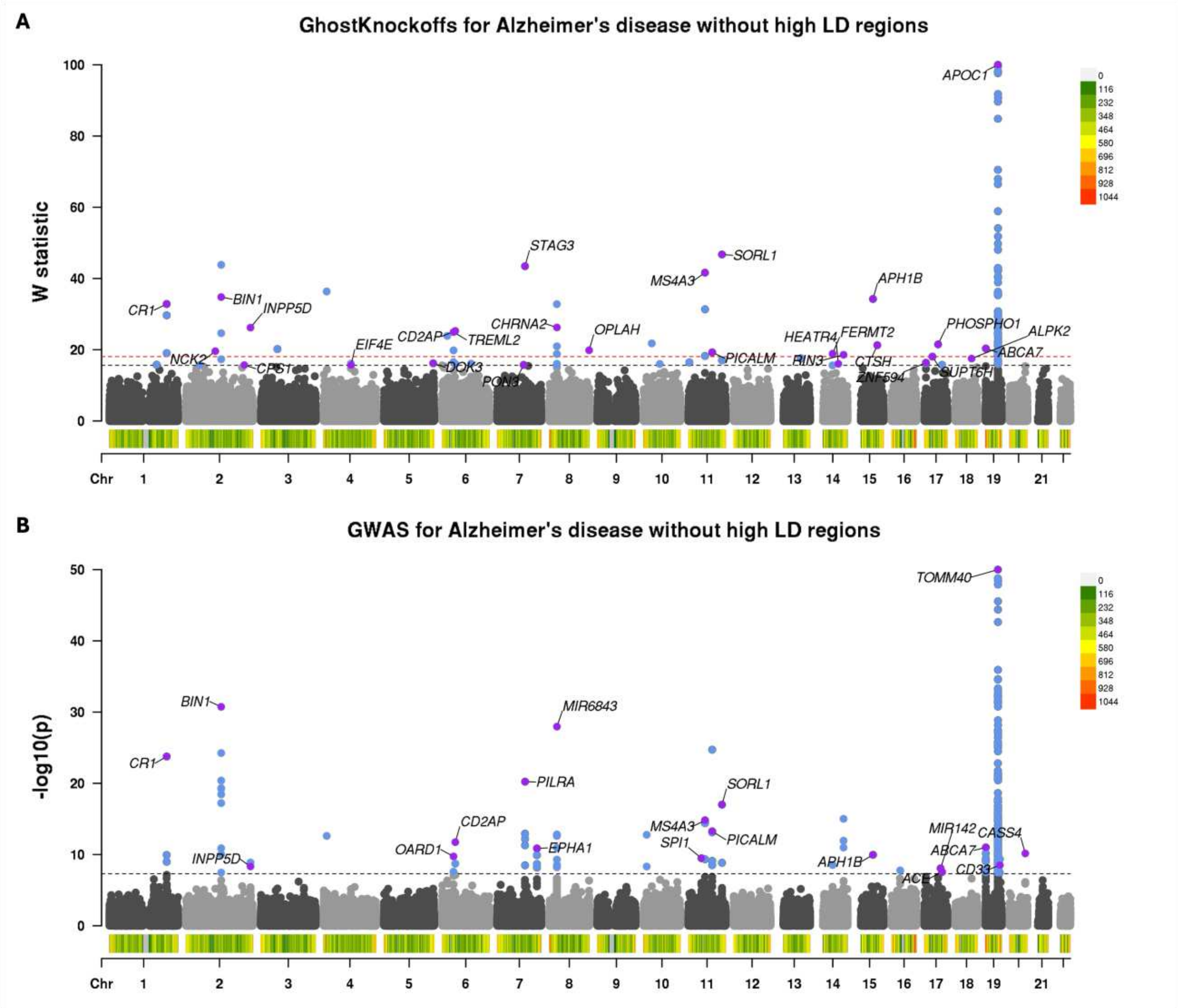

Figure S8: Single-cell transcriptomics data analysis of AD-related genes identified by V2G strategy. We present the differentially expressed gene (DEG) analysis of single-cell RNA sequences consisting of 143,793 single-nucleus transcriptomes for V2G genes based on GhostKnockoffs at FDR levels 0.05/0.1/0.2 and conventional GWASs variable selection results to validate their cell-to-cell variation between AD cases and controls. Each point denotes a gene identified by the V2G strategy. Its color denotes corresponding cell type. The x-axis denotes the $\log_2$(fold change) of average expression between AD cases and controls (positive value indicates gene's higher expression among AD cases). The y-axis denotes $-\log_{10}$(p-value) based on differential expression testing using *Seurat* with the black horizontal dashed line denoting 0.05 threshold for raw p-values. The purple horizontal dashed line denotes Bonferroni adjusted threshold for raw p-values. **A.** DEG analysis of GhostKnockoffs at FDR level 0.1. **B.** DEG analysis of conventional GWASs. **C.** DEG analysis of GhostKnockoffs at FDR level 0.2. **D.** DEG analysis of GhostKnockoffs at FDR level 0.05.

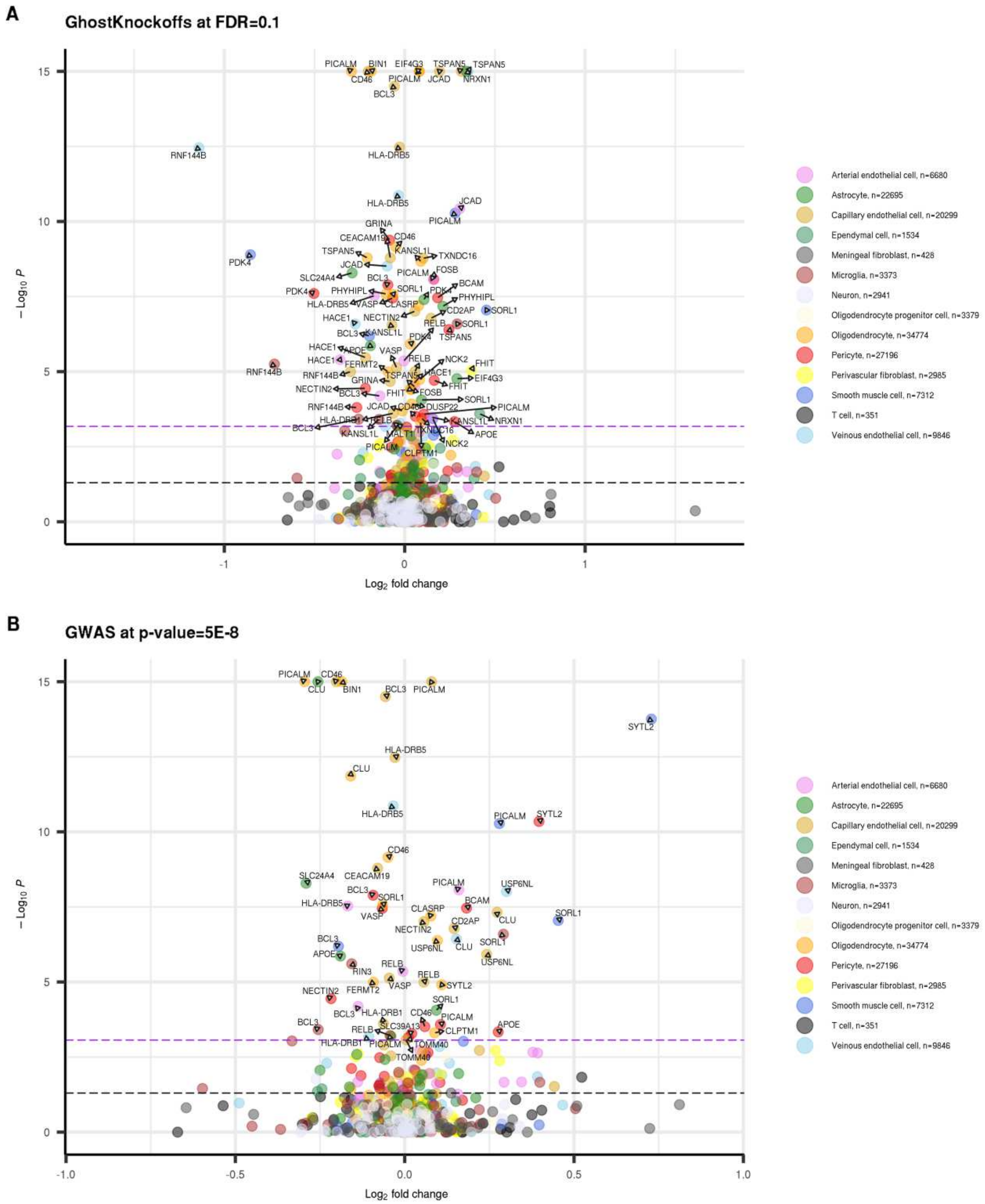

A
GhostKnockoffs at FDR=0.1
B
GWAS at p-value=5E-8
$-\log_{10} P$
$\log_2$ fold change
Arterial endothelial cell, n=6680
Astrocyte, n=22695
Capillary endothelial cell, n=20299
Ependymal cell, n=1534
Meningeal fibroblast, n=428
Microglia, n=3373
Neuron, n=2941
Oligodendrocyte progenitor cell, n=3379
Oligodendrocyte, n=34774
Pericyte, n=27196
Perivascular fibroblast, n=2985
Smooth muscle cell, n=7312
T cell, n=351
Veinous endothelial cell, n=9846

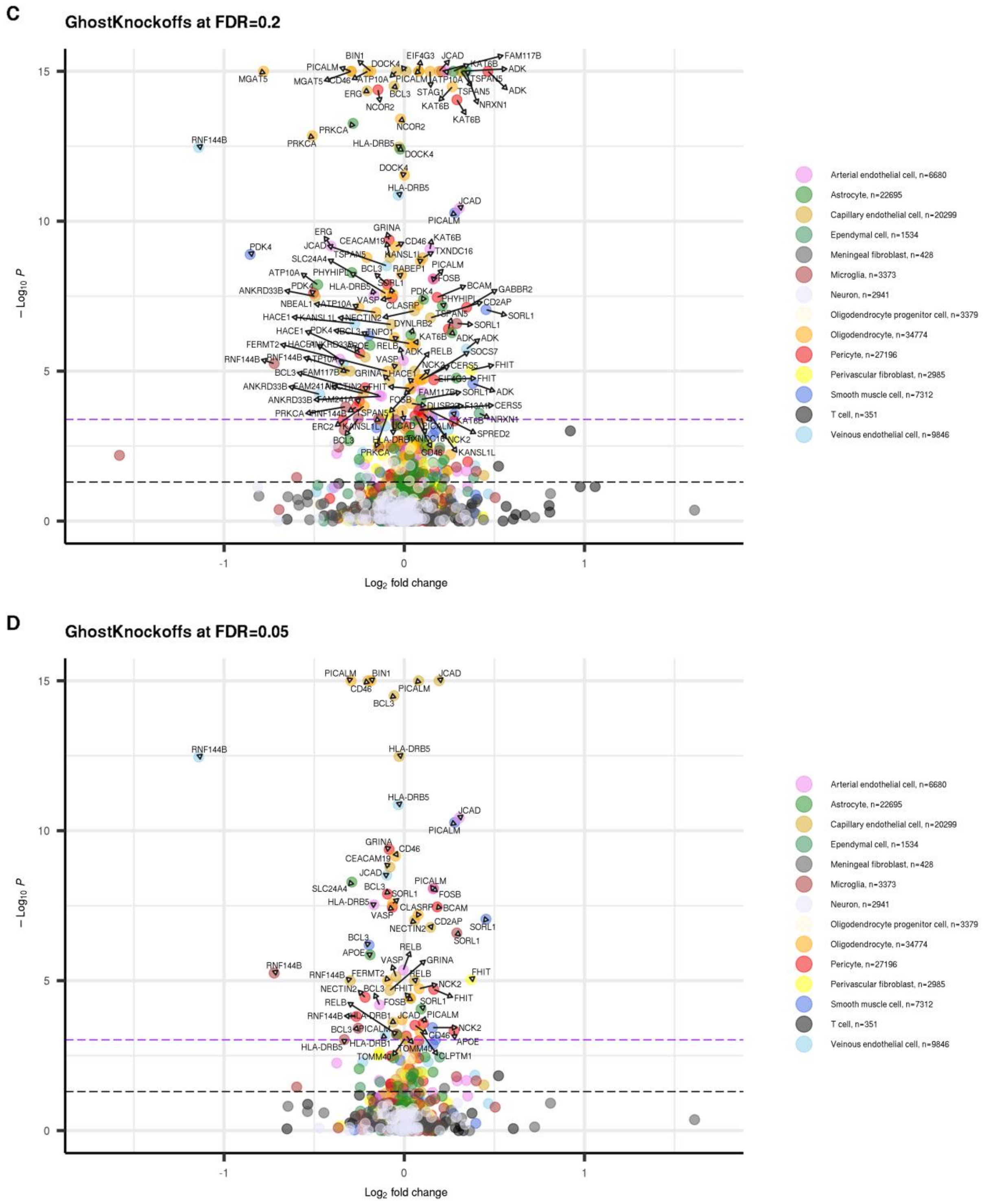
C
GhostKnockoffs at FDR=0.2
$-\log_{10} P$
$\log_2$ fold change
D
GhostKnockoffs at FDR=0.05
$-\log_{10} P$
$\log_2$ fold change
Arterial endothelial cell, n=6680
Astrocyte, n=22695
Capillary endothelial cell, n=20299
Ependymal cell, n=1534
Meningeal fibroblast, n=428
Microglia, n=3373
Neuron, n=2941
Oligodendrocyte progenitor cell, n=3379
Oligodendrocyte, n=34774
Pericyte, n=27196
Perivascular fibroblast, n=2985
Smooth muscle cell, n=7312
T cell, n=351
Veinous endothelial cell, n=9846

Figure S9: Enrichment analysis of single-cell transcriptomics data for identified AD-related genes. We present differentially expressed gene (DEG) analysis of single-cell RNA sequences consisting of 143,793 single-nucleus transcriptomes for AD-related genes identified by the cS2G strategy and proximal genes based on GhostKnockoffs' and conventional GWASs' variable selection results to validate their signal enrichment. **A.** We present proportions of differentially expressed proximal genes stratified by 14 cell types. **B.** We present proportions of DEGs identified by the cS2G strategy stratified by 14 cell types. **C.** We present proportions of DEGs identified by two strategies based on GhostKnockoffs (FDR=0.05/0.1/0.2), GWASs and all background genes. Genes are classified as DEGs if any of the 14 cell types of DE analysis raw p-values is smaller than 0.05.

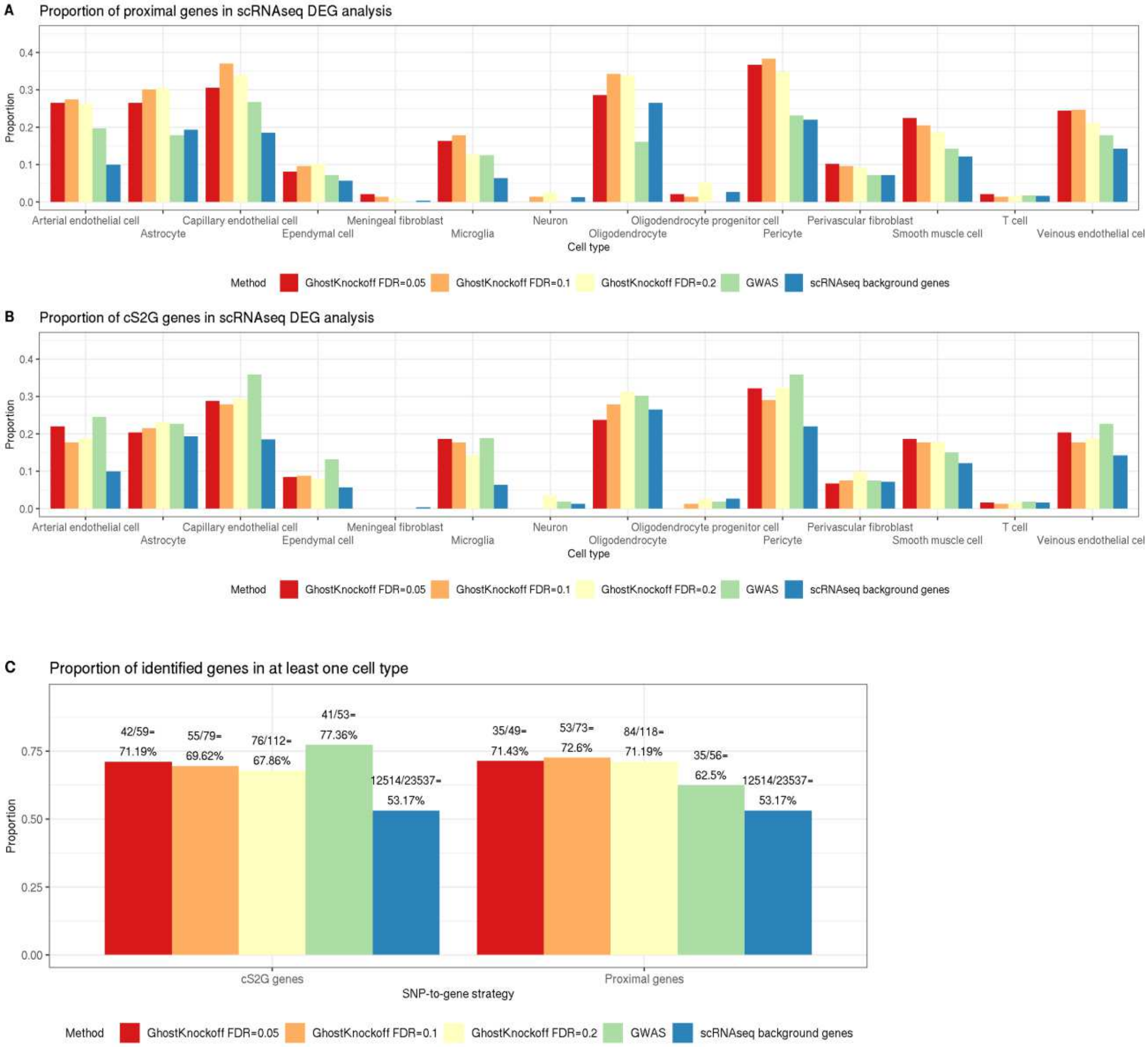

Figure S10: Ranked-based enrichment analysis of single-cell transcriptomics data for identified AD-related genes by locus. We present differentially expressed gene (DEG) analysis of single-cell RNA sequences consisting of 143,793 single-nucleus transcriptomes for AD-related genes identified by the cS2G/V2G strategies and proximal genes based on GhostKnockoffs' and conventional GWASs' variable selection results to validate their signal enrichment. A. We present proportions of DEGs identified by the cS2G strategy stratified by 14 cell types. Genes are classified as DEGs if their DE analysis raw p-values are smaller than 0.05. B. We present proportions of differentially expressed proximal genes stratified by 14 cell types. C. We present proportions of DEGs identified by the V2G strategy stratified by 14 cell types. D. We present proportions of DEGs identified by three based on GhostKnockoffs with mashed number of loci, GWASs and all background genes. Genes are classified as DEGs if any of the 14 cell types of DE analysis raw p-values is smaller than 0.05.

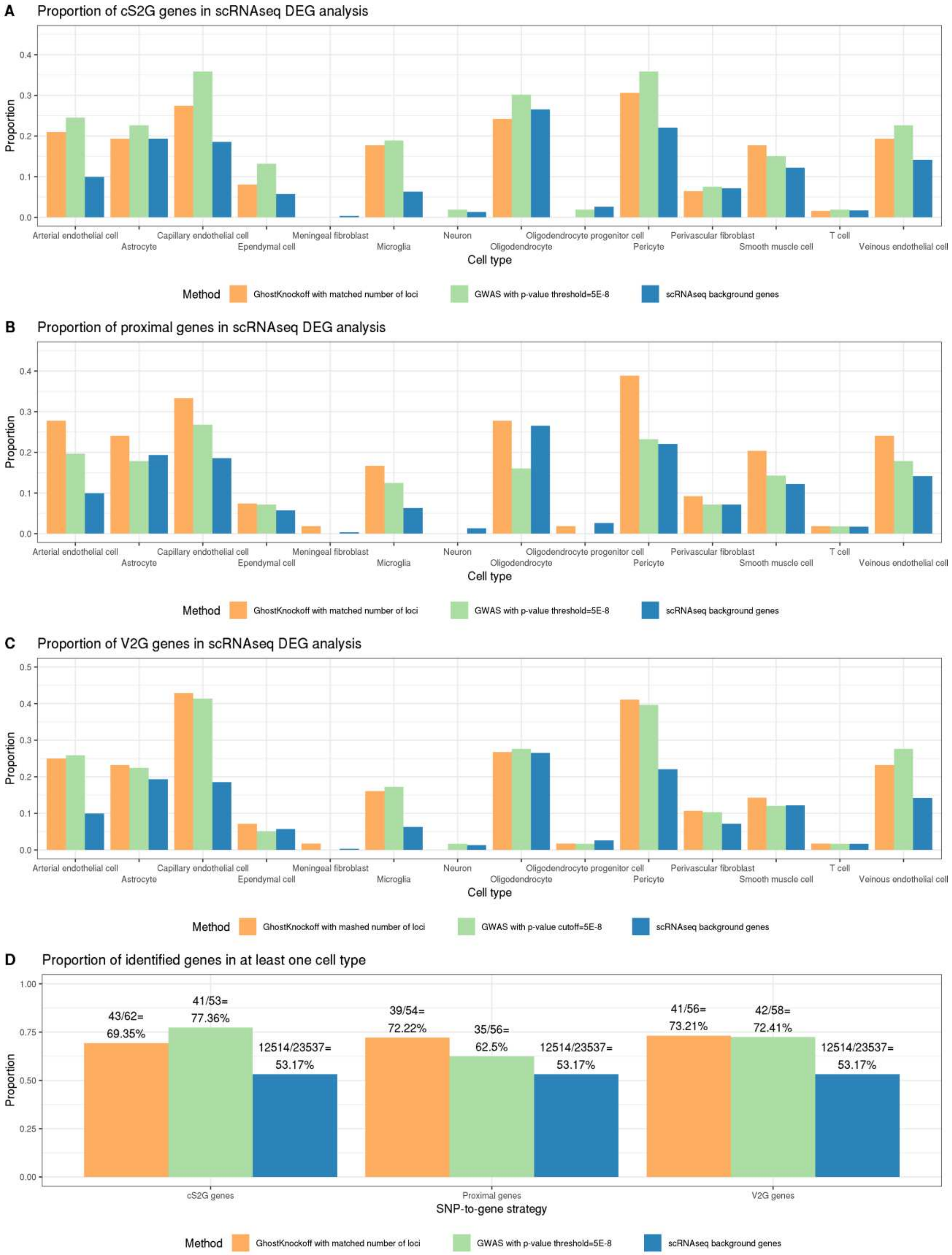
A
Proportion of cS2G genes in scRNAseq DEG analysis
Proportion
0.0
0.1
0.2
0.3
0.4
Arterial endothelial cell
Astrocyte
Capillary endothelial cell
Ependymal cell
Meningeal fibroblast
Microglia
Neuron
Oligodendrocyte
Oligodendrocyte progenitor cell
Pericyte
Perivascular fibroblast
Smooth muscle cell
T cell
Veinous endothelial cell
Cell type
Method
GhostKnockoff with matched number of loci
GWAS with p-value threshold=5E-8
scRNAseq background genes
B
Proportion of proximal genes in scRNAseq DEG analysis
Proportion
0.0
0.1
0.2
0.3
0.4
Arterial endothelial cell
Astrocyte
Capillary endothelial cell
Ependymal cell
Meningeal fibroblast
Microglia
Neuron
Oligodendrocyte
Oligodendrocyte progenitor cell
Pericyte
Perivascular fibroblast
Smooth muscle cell
T cell
Veinous endothelial cell
Cell type
Method
GhostKnockoff with matched number of loci
GWAS with p-value threshold=5E-8
scRNAseq background genes
C
Proportion of V2G genes in scRNAseq DEG analysis
Proportion
0.0
0.1
0.2
0.3
0.4
0.5
Arterial endothelial cell
Astrocyte
Capillary endothelial cell
Ependymal cell
Meningeal fibroblast
Microglia
Neuron
Oligodendrocyte
Oligodendrocyte progenitor cell
Pericyte
Perivascular fibroblast
Smooth muscle cell
T cell
Veinous endothelial cell
Cell type
Method
GhostKnockoff with mashed number of loci
GWAS with p-value cutoff=5E-8
scRNAseq background genes
D
Proportion of identified genes in at least one cell type
Proportion
0.00
0.25
0.50
0.75
1.00
43/62=
69.35%
41/53=
77.36%
12514/23537=
53.17%
39/54=
72.22%
35/56=
62.5%
12514/23537=
53.17%
41/56=
73.21%
42/58=
72.41%
12514/23537=
53.17%
cS2G genes
Proximal genes
V2G genes
SNP-to-gene strategy
Method
GhostKnockoff with matched number of loci
GWAS with p-value threshold=5E-8
scRNAseq background genes

Figure S11: Three-generation pedigree.

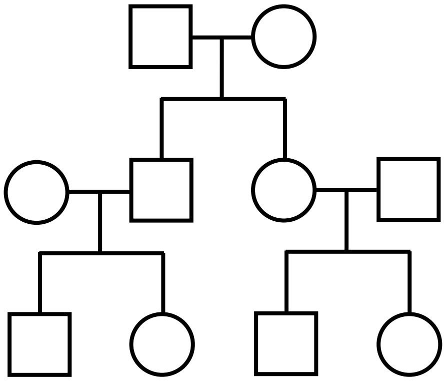

Supplemental Data

Table S1: Independent loci associated with Alzheimer's disease based on GhostKnockoffs at FDR=0.1. For each independent locus, we report its corresponding genetic variant of a hierarchical cluster with the highest GhostKnockoffs W statistic. Given that variants from the same cluster share the same W statistic, we choose the variant with the smallest p-value and its proximal, cS2G and V2G genes to annotate the independent locus. Variants' chromosome numbers and base pair positions are in hg38 coordinates.

Variant	MAF	q	W	p	Proximal gene	cS2G strategy		Open Targets Genetics V2G pipeline			scRNAseq DEG minimum p-value		
						Gene	Mapping info	Gene	pQTL score	eQTL score	Proximal gene	cS2G gene	V2G gene
1:20883276:T:C	0.5277	0.08841	15.94	3.318E-07	EIF4G3	HP1BP3	GTeX_Finemapped	EIF4G3	NA	0.9	2.77E-12	0.00000963	2.77E-12
1:171342675:G:A	0.03575	0.09371	15.78	0.0000512	FMO4	NA	NA	FMO4	NA	0.7	1	NA	1
1:207577223:T:C	0.8012	0.01	32.83	5.528E-29	CR1	CR1	GTeX_Finemapped	CR1	NA	0.8	1	1	1
2:50294490:A:G	0.01017	0.09459	15.71	0.00006184	NRXN1	NA	NA	NRXN1	NA	NA	1.28E-84	NA	1.28E-84
2:105797617:C:T	0.02347	0.03091	19.59	7.155E-07	NCK2	NCK2	EpiMap|ABC	NCK2	NA	0.8	0.4348	0.4348	0.4348
2:127136908:A:T	0.2913	0.01	34.78	9.518E-41	BIN1	BIN1	GTeX_Finemapped	BIN1	NA	0.9	2.657E-13	2.657E-13	2.657E-13
2:210678931:C:T	0.01653	0.09459	15.68	0.00001822	CPS1	CPS1	Exon	KANSL1L	NA	0.5	0.009444	0.009444	0.00005028
2:233117495:T:C	0.9117	0.016	26.2	4.737E-09	INPP5D	INPP5D	EpiMap	INPP5D	NA	0.6	1	1	1
3:59786111:A:C	0.01004	0.02857	20.18	5.929E-06	FHIT	NA	NA	FHIT	NA	NA	0.2175	NA	0.2175
4:11025995:T:C	0.7461	0.01	36.35	1.804E-13	CLNK	NA	NA	CLNK	NA	NA	1	NA	1
4:98608905:C:T	0.01161	0.08421	16.09	0.00002945	TSPAN5	TSPAN5	ABC	TSPAN5	NA	NA	1.365E-41	1.365E-41	1.365E-41
5:177559588:C:G	0.04329	0.08	16.18	1.863E-06	FAM193B	DOK3	eQTLGen_Finemapped	DOK3	NA	0.8	0.5426	1	1
6:219872:C:T	0.07678	0.096	15.57	0.00006847	LOC285766	DUSP22	GTeX_Finemapped	DUSP22	NA	NA	NA	1	1
6:18372310:A:T	0.0444	0.01687	23.88	4.457E-07	RNF144B	NA	NA	RNF144B	NA	0.6	8.202E-09	NA	8.202E-09
6:32610196:T:G	0.1552	0.005556	64.93	4.415E-16	HLA-DRB1	HLA-DRB5	ABC	HLA-DQA2	0.8	1	1	7.851E-09	1
6:41187262:G:A	0.3175	0.01687	24.95	6.926E-08	TREML2	TREML2	EpiMap	NFYA	NA	0.9	NA	NA	1
6:47464901:C:T	0.645	0.01687	25.28	1.679E-14	CD2AP	CD2AP	GTeX_Finemapped	CD2AP	NA	0.7	0.003926	0.003926	0.003926
6:104636049:C:T	0.04696	0.08421	16.09	0.00003523	HACE1	NA	NA	HACE1	NA	NA	0.006099	NA	0.006099

7:95441681:C:T	0.01161	0.09371	15.74	0.0000461	PON2	PON3	EpiMap	PDK4	NA	0.4	1.432E-40	1	0.00003017
7:100179799:G:A	0.2364	0.01	43.44	2.476E-16	STAG3	STAG3	GTeX_Finemapped	STAG3	NA	0.9	1	1	1
8:27365825:T:C	0.5024	0.01	32.79	5.412E-09	PTK2B	PTK2B	EpiMap|ABC	PTK2B	NA	0.9	1	1	1
8:144053248:G:A	0.04783	0.03	19.84	2.511E-07	OPLAH	OPLAH	Exon	GRINA	NA	0.7	1	1	9.823E-06
10:29966853:G:A	0.01791	0.02366	21.78	2.888E-06	JCAD	NA	NA	JCAD	NA	NA	3.855E-51	NA	3.855E-51
10:59033463:G:T	0.01561	0.08613	16	0.00004731	LINC00844	NA	NA	PHYHIPL	NA	NA	1	NA	0.0006104
11:3655238:C:A	0.01679	0.07244	16.44	0.0000259	ART1	NA	NA	ART5	NA	0.4	1	NA	1
11:60079021:A:G	0.275	0.01	41.64	1.322E-15	MS4A3	MS4A3	EpiMap	MS4A6A	NA	1	NA	NA	1
11:86152727:T:G	0.2039	0.03091	19.27	5.24E-10	PICALM	PICALM	EpiMap	PICALM	NA	0.2	3.962E-18	3.962E-18	3.962E-18
11:121564878:T:C	0.03893	0.01	46.73	9.982E-18	SORL1	SORL1	GTeX_Finemapped	SORL1	NA	NA	0.000636	0.000636	0.000636
13:80658153:A:C	0.05201	0.05042	17.6	6.826E-06	SPRY2	NA	NA	SPRY2	NA	NA	1	NA	1
14:52933911:T:C	0.09511	0.03091	18.9	2.653E-10	FERMT2	FERMT2	EpiMap	FERMT2	NA	NA	0.239	0.239	0.239
14:73510230:T:C	0.3595	0.08593	16.04	0.00002006	RIOX1	HEATR4	GTeX_Finemapped| eQTLGen_Finemapped	ACOT1	NA	1	1	1	1
14:92470346:G:A	0.806	0.03333	18.58	3.169E-14	SLC24A4	RIN3	ABC	SLC24A4	NA	1	0.0001214	0.05827	0.0001214
15:63277703:C:T	0.124	0.01	34.23	1.096E-10	APH1B	APH1B	Exon|GTeX_Finemapped| eQTLGen_Finemapped| ABC	APH1B	NA	1	1	1	1
15:78939136:T:C	0.1343	0.02366	21.28	2.896E-06	CTSH	CTSH	Exon|GTeX_Finemapped| EpiMap|ABC	CTSH	1	1	1	1	1
17:5215128:C:T	0.1408	0.075	16.34	0.00002805	SCIMP	ZNF594	GTeX_Finemapped	SCIMP	NA	0.9	1	0.3646	1
17:28698389:G:A	0.0142	0.04576	18.06	0.000011	SUPT6H	SUPT6H	Promoter	RSKR	NA	NA	1	1	NA
17:49229467:C:T	0.2394	0.02366	21.49	1.648E-06	PHOSPHO1	PHOSPHO1	Promoter| GTeX_Finemapped	GNGT2	NA	0.8	1	1	1
17:63480412:A:G	0.01843	0.09444	15.73	1.393E-07	ACE	NA	NA	ACE	NA	NA	1	NA	1
18:58522227:T:C	0.01374	0.05333	17.52	2.374E-07	ALPK2	ALPK2	EpiMap	MALT1	NA	NA	1	1	1
19:1040766:A:G	0.5077	0.02857	20.33	4.111E-11	ABCA7	ABCA7	Promoter|ABC|Cicero	CNN2	NA	0.9	1	1	1
19:44910319:C:T	0.1957	0.005556	1555	0	APOE	SNRPD2	GTeX_Finemapped	NECTIN2	NA	0.8	0.03234	1	0.002291

Table S2: Enrichment analysis of single-cell transcriptomics data for AD-related genes. We report show detailed counts and percentages of differentially expressed genes identified by cS2G strategy based on GhostKnockoffs' and conventional GWASs' variable selection results.

**cS2G strategy**	GWASs (cutoff p-value 5e-8)	GhostKnockoffs FDR=0.1	All background genes
# of cS2G genes	68	99	23537
# of genes with expression measurements in scRNAseq data	53 (53/68=77.94%)	79 (79/99=79.8%)	23537
# of genes with expression & adjusted p-value<0.05	13 (13/53=24.53%)	15 (15/79=18.99%)	3834 (3834/23537=16.29%)
# of genes with expression & raw p-value<0.05	41 (41/53=77.36%)	55 (55/79=69.62%)	12514 (12514/23537=53.17%)

Table S3: Enrichment analysis of single-cell transcriptomics data for AD-related genes. We report show detailed counts and percentages of differentially expressed proximal genes based on GhostKnockoffs' and conventional GWASs' variable selection results.

**Proximal genes**	GWASs (cutoff p-value 5e-8)	GhostKnockoffs FDR=0.1	All background genes
# of proximal genes	73	95	23537
# of genes with expression measurements in scRNAseq data	56 (56/73=76.71%)	73 (73/95=76.84%)	23537
# of genes with expression & adjusted p-value<0.05	11 (11/56=19.64%)	24 (24/73=32.88%)	3834 (3834/23537=16.29%)
# of genes with expression & raw p-value<0.05	35 (35/56=62.5%)	53 (53/73=72.6%)	12514 (12514/23537=53.17%)

Table S4: Enrichment analysis of single-cell transcriptomics data for AD-related genes. We report show detailed counts and percentages of differentially expressed genes identified by V2G strategy based on GhostKnockoffs' and conventional GWASs' variable selection results.

**V2G genes**	GWASs (cutoff p-value 5e-8)	GhostKnockoffs FDR=0.1	All background genes
# of V2G genes	67	82	23537
# of genes with expression measurements in scRNAseq data	58 (58/67=86.57%)	75 (75/82=91.46%)	23537
# of genes with expression & adjusted p-value<0.05	17 (17/58=29.31%)	26 (26/75=34.67%)	3834 (3834/23537=16.29%)
# of genes with expression & raw p-value<0.05	42 (42/58=72.41%)	58 (58/75=77.33%)	12514 (12514/23537=53.17%)

Supplemental Materials

Logistic mixed effect model and score test

For single variant association test (one genetic variant is considered in the model), to test for the genotype effect β with null hypothesis H_0: $\beta = 0$, we consider the following logistic mixed effect model:

$$\text{logit}(\pi_i) = \boldsymbol{X_i \alpha} + G_i\beta + b_i,$$

where $\pi_i = P(y_i = 1|\boldsymbol{X_i}, G_i, b_i)$ denotes the probability of the ith dichotomous phenotype, conditional on covariates $\boldsymbol{X_i}$, genotype G_i and random effect b_i. The random effects are subject to a multivariate Gaussian distribution $\boldsymbol{b} \sim N(\boldsymbol{0}, \theta\boldsymbol{\Phi})$, where θ denotes variance component parameter and $\boldsymbol{\Phi}$ denotes the kinship matrix. With sample size n, the column vector $\boldsymbol{Y} = (Y_1, \cdots, Y_i, \cdots, Y_n)^T$ denotes phenotypes and the column vector $\boldsymbol{G} = (G_1, \cdots, G_i, \cdots, G_n)^T$ denotes the single variant genotypes.

The logistic mixed effect model can be rewritten as:

$$\xi_i = g(\pi_i) = g(\mu_i) = \boldsymbol{X_i^T \alpha} + G_i\beta + b_i,$$

with canonical link function $\xi_i = g(\pi_i) = \log\left(\frac{\pi_i}{1-\pi_i}\right)$. For dichotomous phenotype y_i , $\mu_i = \pi_i = P(y_i = 1|\boldsymbol{X_i}, G_i, b_i)$.

Given the random effect $\boldsymbol{b}$, the dichotomous phenotype y_i is conditionally independent with mean and variance:

$$E(y_i|\boldsymbol{b}) = \mu_i$$

$$Var(y_i|\boldsymbol{b}) = \phi\mu_i(1 - \mu_i),$$

where ϕ denotes the dispersion parameter and $\phi = 1$ for logistic mixed effect model.

For the ith individual, given random effects $\boldsymbol{b}$, the quasi-likelihood of fixed covariate effect $\boldsymbol{\alpha}$ and fixed genotype effect β is:

$$ql_i(\boldsymbol{\alpha}, \beta; \boldsymbol{b}) = \int_{y_i}^{\mu_i} \frac{y_i - \mu}{\mu(1-\mu)} d\mu.$$

The log quasi-likelihood of $(\boldsymbol{\alpha}, \beta, \phi, \theta)$ integrated over the domain of random effect $\boldsymbol{b} \sim N(\boldsymbol{0}, \theta\boldsymbol{\Phi})$ is:

$$ql(\boldsymbol{\alpha}, \beta, \phi, \theta) = \log \int_{\boldsymbol{b}} \exp\left\{\sum_{i=1}^{n} ql_i(\boldsymbol{\alpha}, \beta; \boldsymbol{b})\right\} \times (2\pi)^{-\frac{n}{2}} |\theta\boldsymbol{\Phi}|^{-\frac{1}{2}} \times \exp\left\{-\frac{1}{2}\boldsymbol{b}^T(\theta\boldsymbol{\Phi})^{-1}\boldsymbol{b}\right\} d\boldsymbol{b}.$$

We define the function for simplicity:

$$f(\boldsymbol{b}) = \sum_{i=1}^{n} ql_i(\boldsymbol{\alpha}, \beta; \boldsymbol{b}) - \frac{1}{2}\boldsymbol{b}^T(\theta\boldsymbol{\Phi})^{-1}\boldsymbol{b}.$$

Using the Laplace's method to approximate the above function's integral, we have:

$$\int_{\boldsymbol{b}} \exp\{f(\boldsymbol{b})\}\, d\boldsymbol{b} \approx (2\pi)^{\frac{n}{2}} |-f''(\boldsymbol{b_0})|^{-\frac{1}{2}} \exp\{f(\boldsymbol{b_0})\},$$

where $\boldsymbol{b_0}$= $\text{argmax}_{\boldsymbol{b}} f(\boldsymbol{b})$ achieves $f(\boldsymbol{b})$'s global maximum at $\boldsymbol{b_0}$, and is the solution of $f'(\boldsymbol{b}) = 0$. In this case, the log quasi-likelihood is:

$$ql(\boldsymbol{\alpha}, \beta, \phi, \theta) \approx -\frac{1}{2}\log|\theta\boldsymbol{\Phi}| - \frac{1}{2}\log|-f''(\boldsymbol{b_0})| + f(\boldsymbol{b_0}).$$

To approximate the log quasi-likelihood $ql(\boldsymbol{\alpha}, \beta, \phi, \theta)$, we need to calculate the second partial derivative of $ql_i(\boldsymbol{\alpha}, \beta; \boldsymbol{b})$ with respect to random effects $\boldsymbol{b}$. The first partial derivative of $ql_i(\boldsymbol{\alpha}, \beta; \boldsymbol{b})$ with respect to $\boldsymbol{b}$ is:

$$\frac{\partial ql_i}{\partial \boldsymbol{b}} = \frac{\partial ql_i}{\partial \mu_i}\frac{\partial \mu_i}{\partial \xi_i}\frac{\partial \xi_i}{\partial \boldsymbol{b}} = \frac{y_i - \mu_i}{\mu_i(1-\mu_i)} \cdot \mu_i(1-\mu_i) \cdot \mathbb{I}_i^T = (y_i - \mu_i) \cdot \mathbb{I}_i^T,$$

where $g'(\mu_i) = \frac{1}{\mu_i(1-\mu_i)}$ and $\mathbb{I}_i$ is a $1 \times n$ row vector of indicators such that $b_i = \mathbb{I}_i\boldsymbol{b}$.

The second partial derivative of $ql_i(\boldsymbol{\alpha}, \beta; \boldsymbol{b})$ with respect to $\boldsymbol{b}$ is:

$$\frac{\partial^2 ql_i}{\partial \boldsymbol{b} \partial \boldsymbol{b}^T} = \frac{\partial(y_i - \mu_i)}{\partial \xi_i}\frac{\partial \xi_i}{\partial \boldsymbol{b}^T}\mathbb{I}_i^T + (y_i - \mu_i)\frac{\partial \mathbb{I}_i^T}{\partial \boldsymbol{b}^T} = -\mu_i(1-\mu_i)\mathbb{I}_i^T\mathbb{I}_i,$$

where the second term equals to 0 for canonical link $g(\mu_i) = \xi_i$ because $g'(\mu_i)v(\mu_i) = 1$. Therefore,

$$ql(\boldsymbol{\alpha}, \beta, \phi, \theta) \approx -\frac{1}{2}\log|\theta\boldsymbol{\Phi}| - \frac{1}{2}\log\left|\sum_{i=1}^{n} \mu_i(1-\mu_i)\mathbb{I}_i^T\mathbb{I}_i + (\theta\boldsymbol{\Phi})^{-1}\right| + \sum_{i=1}^{n} ql_i(\boldsymbol{\alpha}, \beta; \boldsymbol{b_0})$$

$$-\frac{1}{2}\boldsymbol{b_0}^T(\theta\boldsymbol{\Phi})^{-1}\boldsymbol{b_0}$$

$$= -\frac{1}{2}\log|\theta\boldsymbol{\Phi} \times (diag(\mu_i(1-\mu_i) + (\theta\boldsymbol{\Phi})^{-1})| + \sum_{i=1}^{n} ql_i(\boldsymbol{\alpha}, \beta; \boldsymbol{b_0}) - \frac{1}{2}\boldsymbol{b_0}^T(\theta\boldsymbol{\Phi})^{-1}\boldsymbol{b_0}$$

$$= -\frac{1}{2}\log\left|\theta\boldsymbol{\Phi} \times diag\big(\mu_i(1-\mu_i)\big) + \boldsymbol{I}\right| + \sum_{i=1}^{n} ql_i(\boldsymbol{\alpha}, \beta; \boldsymbol{b_0}) - \frac{1}{2}\boldsymbol{b_0}^T(\theta\boldsymbol{\Phi})^{-1}\boldsymbol{b_0}.$$

We assume that the weight matrix $diag(\mu_i(1-\mu_i))$ changes slowly with respect to the conditional mean:

$$\frac{\partial diag(\mu_i(1-\mu_i))}{\partial \mu_i} \approx 0.$$

The partial derivatives of $ql(\boldsymbol{\alpha}, \beta, \phi, \theta)$ are:

$$\frac{\partial ql(\boldsymbol{\alpha}, \beta, \phi, \theta)}{\partial \boldsymbol{\alpha}} = \sum_{i=1}^{n} \frac{\partial ql_i}{\partial \mu_i}\frac{\partial \mu_i}{\partial \xi_i}\frac{\partial \xi_i}{\partial \boldsymbol{\alpha}} = \sum_{i=1}^{n} \frac{y_i - \mu_i}{\mu_i(1-\mu_i)} \cdot \mu_i(1-\mu_i) \cdot \boldsymbol{X_i^T} = \boldsymbol{X}^T(\boldsymbol{Y} - \boldsymbol{\mu})$$

$$\frac{\partial ql(\boldsymbol{\alpha}, \beta, \phi, \theta)}{\partial \beta} = \sum_{i=1}^{n} \frac{\partial ql_i}{\partial \mu_i}\frac{\partial \mu_i}{\partial \xi_i}\frac{\partial \xi_i}{\partial \beta} = \sum_{i=1}^{n} \frac{y_i - \mu_i}{\mu_i(1-\mu_i)} \cdot \mu_i(1-\mu_i) \cdot G_i = \boldsymbol{G}^T(\boldsymbol{Y} - \boldsymbol{\mu})$$

$$\frac{\partial ql(\boldsymbol{\alpha}, \beta, \phi, \theta)}{\partial \boldsymbol{b}} = \sum_{i=1}^{n} \frac{\partial ql_i}{\partial \mu_i}\frac{\partial \mu_i}{\partial \xi_i}\frac{\partial \xi_i}{\partial \boldsymbol{b}} - (\theta\boldsymbol{\Phi})^{-1}\boldsymbol{b} = \sum_{i=1}^{n} (y_i - \mu_i) \cdot \mathbb{I}_i^T - (\theta\boldsymbol{\Phi})^{-1}\boldsymbol{b} = (\boldsymbol{Y} - \boldsymbol{\mu}) - (\theta\boldsymbol{\Phi})^{-1}\boldsymbol{b}.$$

Under the null hypothesis $H_0: \beta = 0$, and estimates of dispersion parameter ϕ, kinship matrix $\boldsymbol{\Phi}$ and variance component parameter θ are known, the fixed covariate effect $\widehat{\boldsymbol{\alpha}}(\phi, \theta)$ and random effects $\widehat{\boldsymbol{b}}(\phi, \theta, \boldsymbol{\Phi})$ are estimated simultaneously by maximizing the log quasi-likelihood $ql(\boldsymbol{\alpha}, \beta, \phi, \theta)$. Therefore, $\widehat{\boldsymbol{b}}(\phi, \theta, \boldsymbol{\Phi}) = \boldsymbol{b_0}(\widehat{\boldsymbol{\alpha}}(\phi, \theta), \beta = 0)$ because at $\boldsymbol{b_0}$, $ql(\boldsymbol{\alpha}, \beta, \phi, \theta)$ achieves global maximum.

The working vector of dichotomous phenotypes is $\widetilde{\boldsymbol{Y}} = \left(\tilde{Y}_1, \cdots, \tilde{Y}_n\right)^T$, where $\widetilde{\boldsymbol{Y}} - \boldsymbol{\xi} = g'(\boldsymbol{\mu})(\boldsymbol{Y} - \boldsymbol{\mu})$. The solution $(\boldsymbol{\alpha}, \beta)$ of linear system under the null hypothesis is:

$$\begin{cases} \boldsymbol{X}^T(\boldsymbol{Y} - \boldsymbol{\mu}) = 0 \\ (\boldsymbol{Y} - \boldsymbol{\mu}) - (\theta\boldsymbol{\Phi})^{-1}\boldsymbol{b} = \boldsymbol{0} \end{cases}$$

which is equivalent to solving the following equation:

$$\begin{bmatrix} \boldsymbol{X}^T diag(\boldsymbol{\mu}(\boldsymbol{1} - \boldsymbol{\mu}))\boldsymbol{X} & \boldsymbol{X}^T diag(\boldsymbol{\mu}(\boldsymbol{1} - \boldsymbol{\mu})) \\ diag(\boldsymbol{\mu}(\boldsymbol{1} - \boldsymbol{\mu}))\boldsymbol{X} & (\theta\boldsymbol{\Phi})^{-1} + diag(\boldsymbol{\mu}(\boldsymbol{1} - \boldsymbol{\mu})) \end{bmatrix} \begin{bmatrix} \boldsymbol{\alpha} \\ \boldsymbol{b} \end{bmatrix} = \begin{bmatrix} \boldsymbol{X}^T diag(\boldsymbol{\mu}(\boldsymbol{1} - \boldsymbol{\mu}))\widetilde{\boldsymbol{Y}} \\ diag(\boldsymbol{\mu}(\boldsymbol{1} - \boldsymbol{\mu}))\widetilde{\boldsymbol{Y}} \end{bmatrix}$$

Let $\boldsymbol{\Omega} = diag\left(\frac{\boldsymbol{1}}{\boldsymbol{\mu}(\boldsymbol{1} - \boldsymbol{\mu})}\right) + \theta\boldsymbol{\Phi}$ and $\boldsymbol{\Psi} = \boldsymbol{\Omega}^{-1} - \boldsymbol{\Omega}^{-1}\boldsymbol{X}(\boldsymbol{X}^T\boldsymbol{\Omega}^{-1}\boldsymbol{X})^{-1}\boldsymbol{X}^T\boldsymbol{\Omega}^{-1}$ (projection matrix). The solution to maximize the log quasi-likelihood is:

$$\begin{cases} \widehat{\boldsymbol{\alpha}} = (\boldsymbol{X}^T\boldsymbol{\Omega}^{-1}\boldsymbol{X})^{-1}\boldsymbol{X}^T\boldsymbol{\Omega}^{-1}\widetilde{\boldsymbol{Y}} \\ \widehat{\boldsymbol{b}} = (\theta\boldsymbol{\Phi})\boldsymbol{\Omega}^{-1}\left(\widetilde{\boldsymbol{Y}} - \boldsymbol{X}\widehat{\boldsymbol{\alpha}}\right) \end{cases}$$

$$\widetilde{\boldsymbol{Y}} - \widehat{\boldsymbol{\xi}} = \widetilde{\boldsymbol{Y}} - \boldsymbol{X}\widehat{\boldsymbol{\alpha}} - \widehat{\boldsymbol{b}} = \left(\boldsymbol{I} - \theta\boldsymbol{\Phi}\boldsymbol{\Omega}^{-1}\right)\left(\widetilde{\boldsymbol{Y}} - \boldsymbol{X}\widehat{\boldsymbol{\alpha}}\right) = diag\left(\frac{\boldsymbol{1}}{\boldsymbol{\mu}(\boldsymbol{1} - \boldsymbol{\mu})}\right)\boldsymbol{\Omega}^{-1}\left(\widetilde{\boldsymbol{Y}} - \boldsymbol{X}\widehat{\boldsymbol{\alpha}}\right)$$

$$= diag\left(\frac{\boldsymbol{1}}{\boldsymbol{\mu}(\boldsymbol{1} - \boldsymbol{\mu})}\right)\boldsymbol{\Psi}\widetilde{\boldsymbol{Y}},$$

Under the null hypothesis $H_0\colon \beta = 0$ and estimates of parameters $\left(\widehat{\boldsymbol{\alpha}}, \hat{\phi}, \hat{\theta}\right)$, the score test is constructed by plugging in $\left(\widehat{\boldsymbol{\alpha}}, \hat{\phi}, \hat{\theta}, \beta = 0\right)$ in $\frac{\partial ql(\boldsymbol{\alpha}, \beta, \phi, \theta)}{\partial \beta}$ as follows:

$$T = \frac{\partial ql\left(\widehat{\boldsymbol{\alpha}}, \hat{\phi}, \hat{\theta}, \beta = 0\right)}{\partial \beta} = G^T(\boldsymbol{Y} - \widehat{\boldsymbol{\mu}}) = G^T\left(\widetilde{\boldsymbol{Y}} - \widehat{\boldsymbol{\xi}}\right)\frac{1}{g'(\widehat{\boldsymbol{\mu}})} = G^T diag\left(\frac{\boldsymbol{1}}{\widehat{\boldsymbol{\mu}}(\boldsymbol{1} - \widehat{\boldsymbol{\mu}})}\right)\widehat{\boldsymbol{\Psi}}\widetilde{\boldsymbol{Y}} diag\left(\widehat{\boldsymbol{\mu}}(\boldsymbol{1} - \widehat{\boldsymbol{\mu}})\right)$$

$$= G^T\widehat{\boldsymbol{\Psi}}\widetilde{\boldsymbol{Y}},$$

with corresponding variance being:

$$\mathrm{Var}(T|H_0) = E\left\{\frac{\partial ql\left(\widehat{\boldsymbol{\alpha}}, \hat{\phi}, \hat{\theta}, \beta = 0\right)}{\partial \beta}\frac{\partial ql\left(\widehat{\boldsymbol{\alpha}}, \hat{\phi}, \hat{\theta}, \beta = 0\right)}{\partial \beta^T}\right\} = E\left\{G^T\widehat{\boldsymbol{\Psi}}\widetilde{\boldsymbol{Y}}\left(\boldsymbol{G}^T\widehat{\boldsymbol{\Psi}}\widetilde{\boldsymbol{Y}}\right)^T\right\} = G^T\widehat{\boldsymbol{\Psi}}G.$$

Validity of the GhostKnockoffs Procedure

As shown in Equation (1), Z-scores based on single-variant score test p-values,

$$\boldsymbol{Z}_{\boldsymbol{score}} = \left(Z_{score,1}, \dots, Z_{score,p}\right)^{\mathrm{T}},$$

approximately follow the multivariate Gaussian distribution $N(\boldsymbol{\mu}, \boldsymbol{\Sigma}^*)$ whose covariance matrix $\boldsymbol{\Sigma}^*$ is the correlation matrix analogous to $\boldsymbol{G}^T\widehat{\boldsymbol{\Psi}}\boldsymbol{G}$. However, as matrices $\boldsymbol{P}$ and $\boldsymbol{V}$ are defined by the input LD matrix $\boldsymbol{\Sigma}$ in the GhostKnockoff procedure proposed by He et al.[9], theoretical validity of our procedure warrants further investigation.

Sufficient and Necessary Condition for Exchangeability

To verify the theoretical validity, we need to first derive the sufficient and necessary condition for the exchangeability of $\boldsymbol{Z}_{\boldsymbol{score},\mathcal{H}_0}$ (subvector of $\boldsymbol{Z}_{\boldsymbol{score}}$ corresponding to noncausal variants) and its knockoffs counterparts with respect to the mean vector $\boldsymbol{\mu}$ and the covariance matrix $\boldsymbol{\Sigma}^*$ when the input LD matrix $\boldsymbol{\Sigma}$ is used to compute $\boldsymbol{P}$ and $\boldsymbol{V}$. For simplicity, we consider the case with $M = 1$ knockoff counterpart per genetic variant. Following He et al.[9], matrices $\boldsymbol{P}$ and $\boldsymbol{V}$ are computed as

$$\boldsymbol{P} = \boldsymbol{I} - \boldsymbol{D}\boldsymbol{\Sigma}^{-1} \text{ and } \boldsymbol{V} = 2\boldsymbol{D} - \boldsymbol{D}\boldsymbol{\Sigma}^{-1}\boldsymbol{D},$$

where $\boldsymbol{I}$ is a $p \times p$ identity matrix and $\boldsymbol{D} = \mathrm{diag}(s_1, \dots, s_p)$ is a diagonal matrix obtained by solving the convex optimization problem indexed by $\boldsymbol{\Sigma}$,

$$\text{minimize} \sum_{j=1}^{p} \left|1 - s_j\right|, \text{ subject to} \begin{cases} 2\boldsymbol{\Sigma} - \boldsymbol{D} \geq 0, \\ s_j \geq 0, 1 \leq j \leq p. \end{cases}$$

Thus, if we apply $\boldsymbol{P}$ and $\boldsymbol{V}$ in Equation (1) on $\boldsymbol{Z}_{\boldsymbol{score}}$ with the mean vector $\boldsymbol{\mu}$ and the covariance matrix $\boldsymbol{\Sigma}^*$ (let $\boldsymbol{\Sigma}^* = \boldsymbol{\Sigma} + \boldsymbol{\Delta}$), we have the knockoff counterparts $\widetilde{\boldsymbol{Z}}_{\boldsymbol{score}}$ satisfy

$$\mathrm{E}\left(\widetilde{\boldsymbol{Z}}_{\boldsymbol{score}}\right) = \boldsymbol{P}\boldsymbol{\mu}, \qquad \mathrm{Var}\left(\widetilde{\boldsymbol{Z}}_{\boldsymbol{score}}\right) = \boldsymbol{\Sigma} + \boldsymbol{P}\boldsymbol{\Delta}\boldsymbol{P}^T \text{ and } \mathrm{Cov}\left(\widetilde{\boldsymbol{Z}}_{\boldsymbol{score}}, \boldsymbol{Z}_{\boldsymbol{score}}\right) = \boldsymbol{\Sigma} + \boldsymbol{\Delta} - \boldsymbol{D} - \boldsymbol{D}\boldsymbol{\Sigma}^{-1}\boldsymbol{\Delta}.$$

To satisfy the exchangeability between $\boldsymbol{Z}_{\boldsymbol{score},\mathcal{H}_0}$ and its knockoff counterpart, it is required that

1. $\mathrm{E}(\boldsymbol{Z}_{score}) - \mathrm{E}(\widetilde{\boldsymbol{Z}}_{score}) = \boldsymbol{\mu} - \boldsymbol{P\mu} = \boldsymbol{D\Sigma^{-1}\mu}$ is a sparse vector where only predominant components corresponding to causal variants can be nonzero;
2. $\mathrm{Var}(\boldsymbol{Z}_{score}) - \mathrm{Var}(\widetilde{\boldsymbol{Z}}_{score}) = \boldsymbol{\Delta} - \boldsymbol{P\Delta P}^T = \boldsymbol{0}$;
3. $\mathrm{Var}(\boldsymbol{Z}_{score}) - \mathrm{Cov}(\widetilde{\boldsymbol{Z}}_{score}, \boldsymbol{Z}_{score}) = \boldsymbol{D} + \boldsymbol{D\Sigma^{-1}\Delta}$ is a diagonal matrix.

As $\boldsymbol{D}$ is a diagonal matrix, condition 1 is equivalent to "$\boldsymbol{\Sigma^{-1}\mu}$ is a sparse vector where only predominant components corresponding to causal variants can be nonzero". Thus, we have $\boldsymbol{\mu} \in \mathrm{span}(\boldsymbol{\Sigma}_{\cdot,\mathcal{H}_1})$ where span $(\boldsymbol{\Sigma}_{\cdot,\mathcal{H}_1})$ is the linear spanning space of columns of $\boldsymbol{\Sigma}$ corresponding to causal variants.

As $\boldsymbol{D}$ is a diagonal matrix, condition 3 is equivalent to "$\boldsymbol{D\Sigma^{-1}\Delta}$ is a diagonal matrix". Thus, we let $\boldsymbol{D\Sigma^{-1}\Delta} = \boldsymbol{\Lambda} = \mathrm{diag}(\lambda_1, \dots, \lambda_p)$. By plugging in $\boldsymbol{P} = \boldsymbol{I} - \boldsymbol{D\Sigma^{-1}}$, we have condition 2 is equivalent to

$$\boldsymbol{\Delta} - \boldsymbol{P\Delta P}^T = \boldsymbol{\Delta} - (\boldsymbol{I} - \boldsymbol{D\Sigma^{-1}})\boldsymbol{\Delta}(\boldsymbol{I} - \boldsymbol{\Sigma^{-1}D}) = \boldsymbol{\Delta} - \boldsymbol{\Delta} + 2\boldsymbol{\Lambda} - \boldsymbol{\Lambda\Sigma^{-1}D} = \boldsymbol{\Lambda\Sigma^{-1}D} - 2\boldsymbol{\Lambda} = \boldsymbol{0}.$$

In other words, $\boldsymbol{\Lambda\Sigma^{-1}} = 2\boldsymbol{D^{-1}\Lambda}$ is a diagonal matrix, leading to $\boldsymbol{\Lambda} = \boldsymbol{0}$. Thus, we have $\boldsymbol{D\Sigma^{-1}\Delta} = \boldsymbol{0}$. Because $\boldsymbol{D}$ and $\boldsymbol{\Sigma}$ are generally nonsingular, we have $\boldsymbol{\Delta} = \boldsymbol{0}$.

As a result, the necessary condition for the exchangeability of $\boldsymbol{Z}_{score,\mathcal{H}_0}$ and its knockoff counterparts is

$$\boldsymbol{\mu} \in \mathrm{span}(\boldsymbol{\Sigma}_{\cdot,\mathcal{H}_1}) \text{ and } \boldsymbol{\Sigma}^* = \boldsymbol{\Sigma}.$$

In addition, as this condition is also trivially sufficient, it is the sufficient and necessary condition for the exchangeability of $\boldsymbol{Z}_{score,\mathcal{H}_0}$ and its knockoff counterparts.

From Exchangeability to FDR Control

Although we have found the sufficient and necessary condition for the exchangeability of $\boldsymbol{Z}_{score,\mathcal{H}_0}$ and its knockoff counterparts, it is still required to illustrate how such an exchangeability provides valid FDR control. Consider feature statistics,

$$\kappa_j = \begin{cases} 0, & \text{if } (Z_{score,j})^2 = T_j^{max} \\ m, & \text{if } (\tilde{Z}_{score,j}^m)^2 = T_j^{max} \text{ for } m = 1, \dots, M \end{cases}, \qquad \tau_j = T_j^{max} - T_j^{median}, \qquad \text{for } j = 1, \dots, p,$$

where

$$\begin{cases} T_j^{max} = \max\left\{\left(Z_{score,j}\right)^2, \left(\tilde{Z}_{score,j}^1\right)^2, \dots, \left(\tilde{Z}_{score,j}^M\right)^2\right\}, \\ T_j^{median} = \text{median}\left(\left\{\left(Z_{score,j}\right)^2, \left(\tilde{Z}_{score,j}^1\right)^2, \dots, \left(\tilde{Z}_{score,j}^M\right)^2\right\} \setminus \left\{T_j^{max}\right\}\right). \end{cases}$$

Given the exchangeability of $\boldsymbol{Z}_{\boldsymbol{score},\mathcal{H}_0}$ and its knockoff counterparts, the joint distribution of $\boldsymbol{Z}_{\boldsymbol{score}}, \widetilde{\boldsymbol{Z}}_{\boldsymbol{score}}^1, \dots, \widetilde{\boldsymbol{Z}}_{\boldsymbol{score}}^M$ is invariant with respect to any permutation among $Z_{score,j}, \tilde{Z}_{score,j}^1, \dots, \tilde{Z}_{score,j}^M$ for all $j \in \mathcal{H}_0$. In other words, for any permutations $\boldsymbol{\sigma} = \left\{\sigma_1, \dots, \sigma_p\right\}$ where σ_j is an arbitrary permutation on $\{0, \dots, M\}$ if $j \in \mathcal{H}_0$ and the identity permutation otherwise, we have

$$\left(\boldsymbol{Z}_{\boldsymbol{score}}, \widetilde{\boldsymbol{Z}}_{\boldsymbol{score}}^1, \dots, \widetilde{\boldsymbol{Z}}_{\boldsymbol{score}}^M\right)_{\boldsymbol{\sigma}} =^D \left(\boldsymbol{Z}_{\boldsymbol{score}}, \widetilde{\boldsymbol{Z}}_{\boldsymbol{score}}^1, \dots, \widetilde{\boldsymbol{Z}}_{\boldsymbol{score}}^M\right),$$

under the convention that $\widetilde{\boldsymbol{Z}}_{\boldsymbol{score}}^0 = \boldsymbol{Z}_{\boldsymbol{score}}$ and

$$\left(\boldsymbol{Z}_{\boldsymbol{score}}, \widetilde{\boldsymbol{Z}}_{\boldsymbol{score}}^1, \dots, \widetilde{\boldsymbol{Z}}_{\boldsymbol{score}}^M\right)_{\boldsymbol{\sigma}} = \left(\tilde{Z}_{score,1}^{\sigma_1(0)}, \dots, \tilde{Z}_{score,p}^{\sigma_p(0)}, \dots, \tilde{Z}_{score,1}^{\sigma_1(M)}, \dots, \tilde{Z}_{score,p}^{\sigma_p(M)}\right).$$

Because for all j, feature statistics corresponding to $\left(\boldsymbol{Z}_{\boldsymbol{score}}, \widetilde{\boldsymbol{Z}}_{\boldsymbol{score}}^1, \dots, \widetilde{\boldsymbol{Z}}_{\boldsymbol{score}}^M\right)_{\boldsymbol{\sigma}}$ (denoted as $\kappa_j^{\boldsymbol{\sigma}}$ and $\tau_j^{\boldsymbol{\sigma}}$ for $j = 1, \dots, p$) satisfy that

$$\kappa_j^{\boldsymbol{\sigma}} = \sigma_j^{-1}\left(\kappa_j\right) \text{ and } \tau_j^{\boldsymbol{\sigma}} = \tau_j, \qquad \text{for } j = 1, \dots, p,$$

we have

$$\left(\sigma_1^{-1}(\kappa_1), \tau_1, \dots, \sigma_p^{-1}\left(\kappa_p\right), \tau_p\right) =^D \left(\kappa_1, \tau_1, \dots, \kappa_p, \tau_p\right).$$

Since σ_j is the identity permutation for all $j \notin \mathcal{H}_0$, we have

$$\left(\left\{\sigma_j^{-1}\left(\kappa_j\right)\right\}_{j\in\mathcal{H}_0}, \left\{\kappa_j\right\}_{j\notin\mathcal{H}_0}, \left\{\tau_j\right\}_{j=1,\dots,p}\right) =^D \left(\left\{\kappa_j\right\}_{j\in\mathcal{H}_0}, \left\{\kappa_j\right\}_{j\notin\mathcal{H}_0}, \left\{\tau_j\right\}_{j=1,\dots,p}\right)$$

and thus

$$\left\{\sigma_j^{-1}\left(\kappa_j\right)\right\}_{j\in\mathcal{H}_0} \Big| \left\{\kappa_j\right\}_{j\notin\mathcal{H}_0}, \left\{\tau_j\right\}_{j=1,\dots,p} =^D \left\{\kappa_j\right\}_{j\in\mathcal{H}_0} \Big| \left\{\kappa_j\right\}_{j\notin\mathcal{H}_0}, \left\{\tau_j\right\}_{j=1,\dots,p}.$$

Because σ_j is an arbitrary permutation on $\{0, \dots, M\}$ for all $j \in \mathcal{H}_0$, σ_j^{-1} is an arbitrary permutation. As a result, κ_j's for all noncausal variants ($\mathcal{H}_0$) are i.i.d. distributed uniformly on $0, \dots, M$ conditional on all τ_j's and κ_j's for all causal variants,

$$\{\kappa_j\}_{j \in \mathcal{H}_0} \Big| \{\kappa_j\}_{j \notin \mathcal{H}_0}, \{\tau_j\}_{j=1,\dots,p} \sim \mathrm{Unif}\left(\{0, \dots, M\}^{|\mathcal{H}_0|}\right).$$

Following Proposition 3.3 of Gimenez et al.[32], our procedure with feature statistics $\{(\kappa_j, \tau_j)\}_{j=1,\dots,p}$ can provide valid FDR control for any target level $\alpha \in (0,1)$.
